# Fluid-electrolyte homeostasis requires histone deacetylase function

**DOI:** 10.1172/jci.insight.137792

**Published:** 2020-08-20

**Authors:** Kelly A. Hyndman, Joshua S. Speed, Luciano D. Mendoza, John M. Allan, Jackson Colson, Randee Sedaka, Chunhua Jin, Hyun Jun Jung, Samir El-Dahr, David M. Pollock, Jennifer S. Pollock

**Affiliations:** 1 Section of Cardio-Renal Physiology and Medicine, Division of Nephrology, Department of Medicine, University of Alabama at Birmingham, Birmingham, Alabama, USA.; 2 Department of Physiology and Biophysics, University of Mississippi Medical Center, Jackson, Mississippi, USA.; 3 Division of Nephrology, Department of Medicine, Johns Hopkins University School of Medicine, Baltimore, Maryland, USA.; 4 Department of Pediatrics, Tulane University School of Medicine, New Orleans, Louisiana, USA.

**Keywords:** Cell Biology, Nephrology, Bioinformatics, Epigenetics, Epithelial transport of ions and water

## Abstract

Histone deacetylase (HDAC) enzymes regulate transcription through epigenetic modification of chromatin structure, but their specific functions in the kidney remain elusive. We discovered that the human kidney expresses class I HDACs. Kidney medulla-specific inhibition of class I HDACs in the rat during high-salt feeding results in hypertension, polyuria, hypokalemia, and nitric oxide deficiency. Three new inducible murine models were used to determine that HDAC1 and HDAC2 in the kidney epithelium are necessary for maintaining epithelial integrity and maintaining fluid-electrolyte balance during increased dietary sodium intake. Moreover, single-nucleus RNA-sequencing determined that epithelial HDAC1 and HDAC2 are necessary for expression of many sodium or water transporters and channels. In performing a systematic review and meta-analysis of serious adverse events associated with clinical HDAC inhibitor use, we found that HDAC inhibitors increased the odds ratio of experiencing fluid-electrolyte disorders, such as hypokalemia. This study provides insight on the mechanisms of potential serious adverse events with HDAC inhibitors, which may be fatal to critically ill patients. In conclusion, kidney tubular HDACs provide a link between the environment, such as consumption of high-salt diets, and regulation of homeostatic mechanisms to remain in fluid-electrolyte balance.

## Abstract 2

Kidney histone deacetylase activity is necessary for maintaining fluid-electrolyte balance during environmental insults such as a high sodium diet.

## Graphical abstract

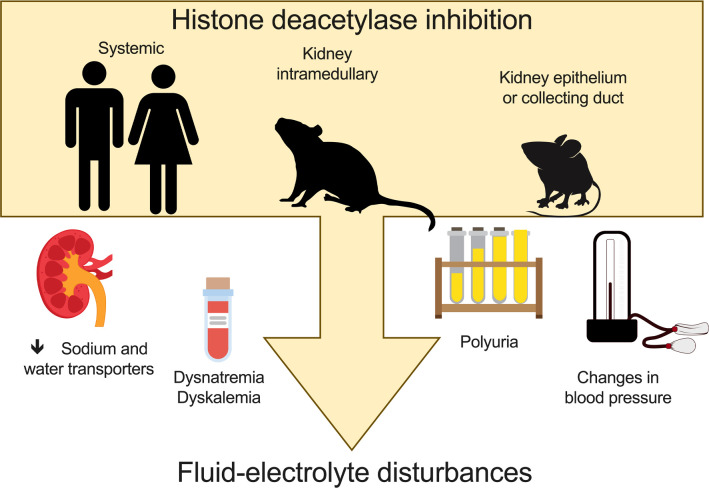

## Introduction

Maintenance of fluid-electrolyte balance during challenges such as high-salt diets involves integration of endocrine, paracrine, and autocrine factors. In normotensive individuals high-salt feeding results in a suppression of antinatriuretic pathways, such as the renin-angiotensin-aldosterone system (RAAS), and activation of natriuretic pathways, such as the collecting duct endothelin-1/nitric oxide (NO) cascades (e.g., ref. 1). These physiological changes result in excretion of excess salt and water and prevent volume expansion and the potential for an increase in blood pressure. However, disruption in these pathways can lead to salt-sensitive changes in blood pressure, even in normotensive patients (2–5). Epidemiological data suggest that 47% of hypertensive patients are on at least 3 classes of antihypertensive drugs but only 60% of treated hypertensive patients have controlled blood pressure (6). Thus, there is a need to elucidate other pathways that are critical for the maintenance of fluid-electrolyte balance.

Histone deacetylases (HDACs) are a large family of enzymes that deacetylate lysine residues of histones to regulate chromatin structure and subsequent gene transcription. The HDAC isoforms are categorized into 4 classes based upon structure: class I (HDAC1, -2, -3, -8), class II (HDAC4, -5, -6, -7, -9, -10), class III (sirtuins 1–11), and class IV (HDAC11) (7). Elevated HDAC activity has been causatively linked to cancer, and consequently there are 4 FDA-approved HDAC inhibitors (HDACi) for the treatment of T cell lymphoma (8). These are the non–class-selective HDACi, vorinostat, belinostat (9), and panobinostat (10), and the class I–selective HDACi, romidepsin (11). As many as 15 additional HDACi (12) are currently registered in more than 500 clinical trials for the treatment of a variety of cancers. Recently, HDACi have been proposed to be beneficial in a number of cardiovascular and renal diseases, including heart failure (13), renal ischemia/reperfusion injury (14), and diabetic nephropathy (15). However, a consistently reported adverse event of treatment with HDACi is hyponatremia (16–19). Hyponatremia affects as many as 24.5% of intensive care unit patients and can lead to life-threatening neurological complications (20). Moreover, other adverse events of HDACi include hypokalemia, dehydration, diarrhea, and limb edema (16–19). These types of fluid-electrolyte disturbances are potentially fatal to critically ill patients, yet the HDACi-mediated mechanisms are not understood.

In the kidney, the distal portion of the nephron is responsive to antinatriuretic or antidiuretic hormones, such as aldosterone and vasopressin. Furthermore, paracrine/autocrine, natriuretic, and diuretic factors, such as NO, are produced at the highest levels in the inner medullary collecting duct (21, 22). Studies with collecting duct principal cell–specific NO system–knockout mice have provided compelling evidence that NO is critical for fluid-electrolyte balance and blood pressure control (23, 24). Thus, HDACs in the renal medulla may be a critical site of regulation of these natriuretic factors. Furthermore, according to the RNA-Seq database of the rat kidney, class I HDACs have the greatest expression in the distal nephron compared with the other nephron segments (25). Ureteric bud HDAC1 and HDAC2 are necessary for proper kidney development (26); however, their role in the adult nephron is unclear. We recently reported that class I HDACs are expressed in the adult murine kidney (27). The purpose of the current study was to test the hypothesis that renal medullary class I HDACs are critical mediators between a change in the environment, such as eating diets high in sodium, and activation of mechanisms to maintain fluid-electrolyte homeostasis. The findings of our study support the concept that chronic use of HDACi or an inability to appropriately activate kidney epithelial HDAC1 and HDAC2 leads to severe fluid-electrolyte disturbances and persistent kidney damage.

## Results

### Class I HDACs are expressed in the renal medulla.

**Figure 1:**
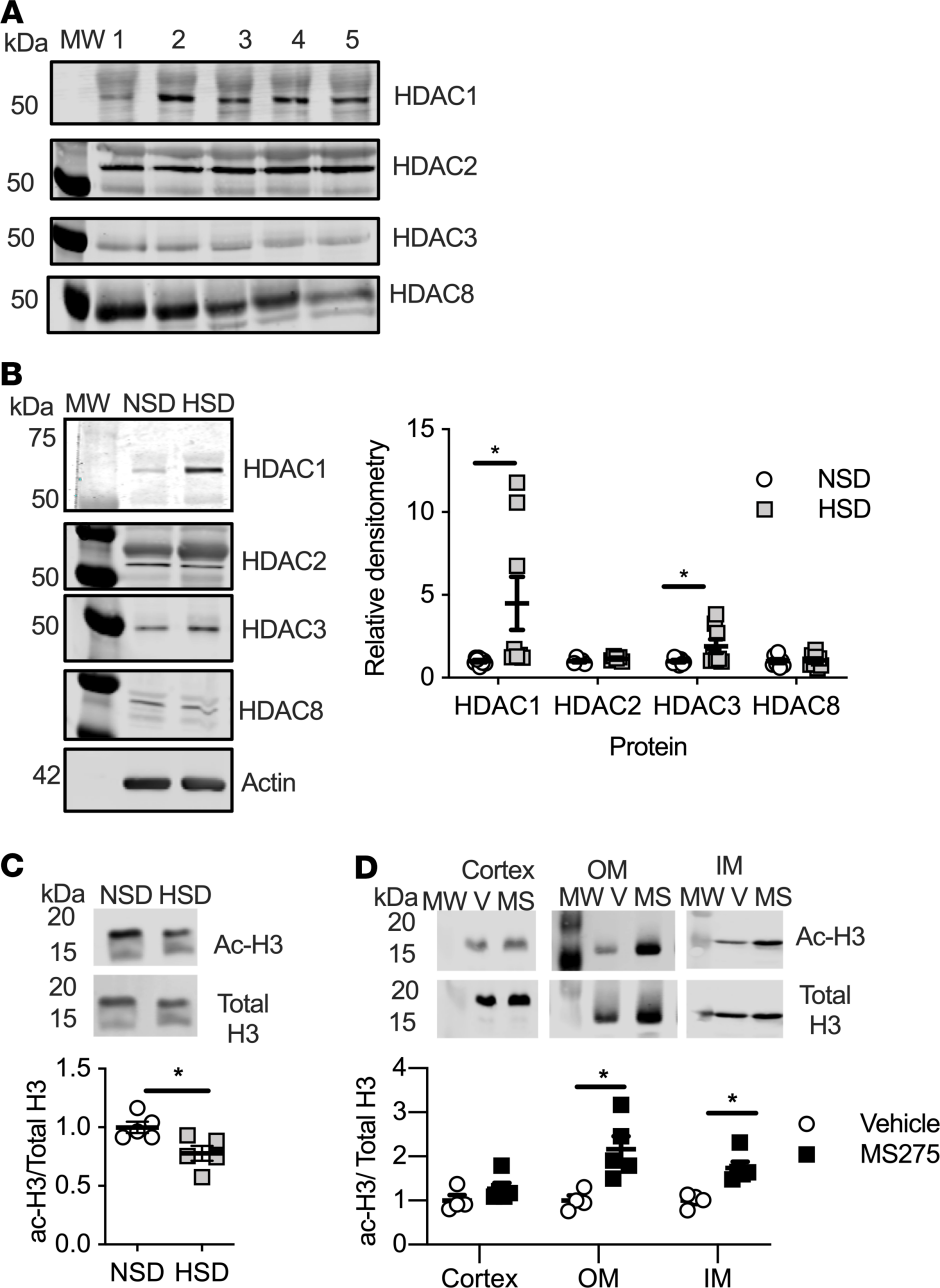
Class I HDACs are expressed in the kidney. (**A**) Human cortical/medullary lysates (*n* = 5 individuals). (**B**) Male Sprague-Dawley rats on a normal (NS, open circles) or 4% high-salt (HS, closed squares) chow diet express class I HDACs in the inner medulla. After 7 days of HS diet (HSD), there was a significant increase in IM protein expression of HDAC1 and HDAC3 (*n* = 8 rats/group, 2-tailed Student’s *t* test, *P < 0.05). (**C**) HSD also results in activation of IM HDAC activity as determined by a decrease in histone H3–lysine acetylation (ac-H3). (**D**) Intramedullary inhibition of class I HDACs with MS275 for 7 days, while on an HSD, results in a significant increase in IM and outer medullary (OM) histone ac-H3 but not in the cortex. (*n* = 4 vehicle, *n* = 5 MS275; 2-tailed Student’s *t* test, *P = 0.01). Individual data points shown with mean ± SEM plotted. MW, molecular weight; V, vehicle.

Western blots were performed with human kidney lysates (mixed sexes, 60% cortex, 40% medulla) and male rat inner medullary (IM) lysates. The class I HDACs (HDAC1, -2, -3, and -8) were expressed in medullary/cortical lysates from 5 human subjects (Figure 1A). Similarly, in the rat IM lysate, all 4 class I HDAC isoforms were expressed (Figure 1B). Next, to determine whether the class I HDACs are regulated by dietary sodium intake, rats were placed on a normal (0.49% NaCl) or high-salt (4.0% NaCl) diet for 7 days. High-salt feeding resulted in a significant 4-fold increase in HDAC1 (*n* = 8/group, *P* = 0.04) and 2-fold increase in HDAC3 IM expression (*P* = 0.04) (Figure 1B). IM HDAC2 and HDAC8 expression were not significantly affected by 7 days of high-salt feeding (*P* > 0.05). A hallmark of HDAC nuclear activity is a decrease in histone H3–lysine acetylation (28). Consistent with increased HDAC activity, there was a significant decrease in IM histone H3–lysine acetylation after 7 days of high-salt feeding compared with IM from normal salt–fed rats (Figure 1C). These data show that both HDAC expression and HDAC activity are increased by high salt intake.

### Intramedullary infusion of MS275, a class I HDAC inhibitor, leads to an increase in blood pressure.

To determine whether medullary class I HDACs are involved in fluid-electrolyte balance and blood pressure control, rats were uninephrectomized, implanted with telemetry transmitters, and a week later implanted with programmable, peristaltic pumps to infuse within the intramedullary region of the remaining kidney. The pumps were filled with vehicle or the class I HDACi MS275. Two separate cohorts of rats were placed on a high-salt intake that was delivered by 2 classic protocols: feeding of HSD or drinking high salt (1% NaCl) in water (HSW) for 7 days. To confirm HDAC inhibition with MS275, histones were extracted from the inner medulla, outer medulla, and cortex of the kidney. Intramedullary MS275 infusion resulted in a significant increase in histone H3-lysine acetylation in the inner medulla and outer medulla (Figure 1D). Histone H3–lysine acetylation in the cortex was similar between vehicle- and MS275-infused rats (Figure 1D), indicating targeted delivery of the drug that was confined to the medulla of the kidney.

During the high salt loading in the rats, inhibition of medullary class I HDACs with MS275 resulted in a significant increase in mean arterial, systolic, and diastolic pressure by day 4 of intramedullary infusion that continued to rise over the 7 days of the study (Supplemental Figure 1 and Supplemental Table 1; supplemental material available online with this article; https://doi.org/10.1172/jci.insight.137792DS1). Similar results were also seen in the HSW study (Supplemental Figure 2 and Supplemental Table 1). As blood pressure increased in the MS275-treated rats, heart rate significantly decreased as expected (Supplemental Figures 1 and 2 and Supplemental Table 1).

### Intramedullary infusion of MS275 results in changes in thirst.

**Figure 2:**
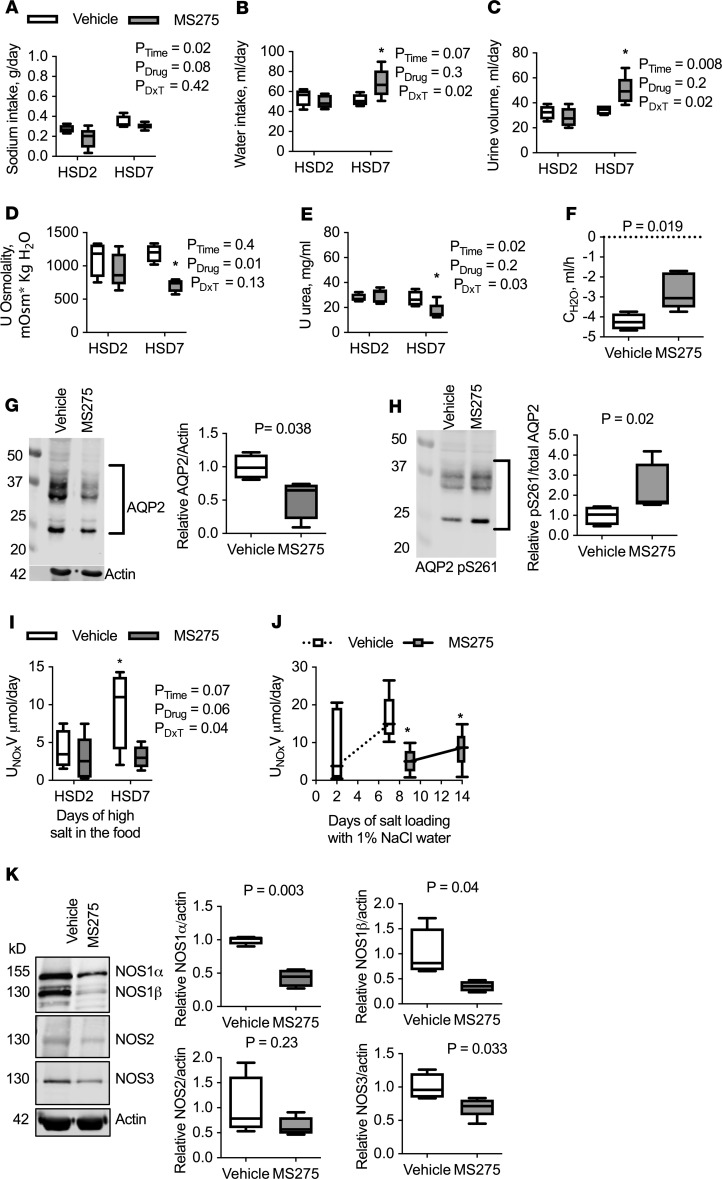
Metabolic cage results from rats on a 2- or 7-day HSD with intramedullary infusion of MS275 (gray) or vehicle (white). Box plots with median and maximum and minimum values plotted. (**A**) MS275 treatment does not significantly affect sodium intake but (**B**) leads to increased consumption of water and (**C**) subsequent increase in urine production after 7 days, compared with vehicle-infused rats. (**D**) Urine osmolality and (**E**) urinary urea concentration were reduced with MS275 treatment. Two-factor ANOVA reported and *P < 0.05 compared with vehicle from post hoc Holm-Šidák multiple comparison test reported. (**F**) Free water clearance (C_H2O_) shows production of a dilute urine; however, it is significantly different with MS275 treatment. Unpaired, 2-tailed Student’s *t* test reported. (**G** and **H**) IM expression of AQP2 and the inhibitory phosphorylation site of AQP2 S261 after 7 days of vehicle or MS275 infusion. (**G**) MS275 treatment results in a significant reduction in AQP2 expression and (**H**) a significant increase in phosphorylation of S261. Unpaired, 2-tailed Student’s *t* test reported. Intramedullary infusion of the class I HDAC inhibitor MS275 results in reduced NO. (**I**) Urinary NOx (nitrite + nitrate) excretion fails to increase in MS275-infused rats that were eating a 2- and 7-day HSD. Two-factor ANOVA and *P < 0.05 compared with vehicle HSD2 from post hoc Holm-Šidák multiple comparison test reported. (**J**) Urinary NOx excretion is significantly decreased with MS275 treatment in rats drinking 1% NaCl. There was a decrease after 2 days of MS275 treatment. *P < 0.05 from vehicle day 7 as determined by paired, 2-tailed Student’s *t* test. (**K**) IM NOS expression in rats on a high-salt chow. Seven days of MS275 treatment results in a significant decrease in NOS1α, NOS1β, and NOS3 protein abundance. NOS2 expression was not statistically significant from vehicle-treated rats. *n* = 4 for vehicle, 5 for MS275. Unpaired, 2-tailed Student’s *t* test reported. DxT, interaction between drug and time.

To determine whether class I HDAC inhibition with MS275 infusion significantly affected fluid-electrolyte balance, rats were placed in metabolic cages and analyzed on day 2 of the study (when blood pressure was similar between the groups) and on day 7 (when there was a significant increase in blood pressure). Sodium intake was similar between vehicle- and MS275-treated rats eating an HSD over the course of the study (Figure 2A and Supplemental Table 2). However, on day 7 of MS275 infusion, rats consumed significantly more water (Figure 2B and Supplemental Table 2), indicating an increase in thirst. Rats that were salt loaded with drinking water and MS275 infusion also presented with significant thirst, consuming approximately 10 mL more than vehicle-infused rats (Supplemental Table 3). To determine whether the kidney was effectively managing this increase in fluid intake, urine output and composition were determined. Salt-loaded rats with MS275 treatment had a significantly higher urine output compared with vehicle-treated rats (Figure 2C). Urine osmolality and urea concentration were significantly reduced with MS275 treatment (Figure 2, D and E; and Supplemental Table 2). Free water clearance was significantly greater in the MS275-infused rats (*P* = 0.019), demonstrating that MS275 resulted in more dilute urine (Figure 2F and Supplemental Table 2). This indicates that medullary class I HDAC inhibition leads to changes in the kidney medulla, resulting in increased thirst during chronic high-salt intake and a subsequent greater volume of fluid that must be efficiently managed by the kidney.

Plasma sodium and chloride concentrations were similar between the rats receiving either vehicle or MS275 intramedullary infusions (Supplemental Table 4). Plasma osmolality was not statistically different between vehicle- and MS275-treated rats (Supplemental Table 4). Plasma potassium was significantly lower in both groups of rats receiving MS275 treatment compared with vehicle (*P* < 0.05; Supplemental Table 4).

Creatinine clearance was similar between the rats receiving vehicle or MS275 infusions, suggesting normal glomerular filtration rate (Supplemental Tables 2 and 3). Potassium excretion was similar among all groups (Supplemental Tables 2 and 3). Sodium excretion in the HSD-fed animals with vehicle or MS275 infusion was similar on both day 2 and day 7 of infusion (Supplemental Table 2). However, HSW rats with MS275 infusion had a significant increase in urinary sodium excretion on day 7 compared with vehicle-treated HSW rats (Supplemental Table 3), yet plasma sodium appeared normal in this group of rats (Supplemental Table 4).

### Intramedullary infusion of MS275 results in altered natriuretic/diuretic regulatory factors.

Fluid-electrolyte balance involves many paracrine/autocrine and endocrine factors that either promote or inhibit natriuresis/diuresis to maintain homeostasis. There was no significant effect of MS275 infusion on urinary atrial natriuretic peptide or aldosterone excretion on day 2 or day 7 of HSD or HSW (Supplemental Tables 2 and 3). Likewise, plasma aldosterone and plasma renin concentration (as an index of RAAS status) were similar among the groups (Supplemental Table 4). Vasopressin excretion was significantly increased 2.5-fold in MS275-infused rats on day 7 of HSD, which agrees with an increase in thirst in these animals (Supplemental Table 2). Moreover, IM aquaporin-2 (AQP2) expression was significantly reduced in MS275-treated rats, and phosphorylation of S261 (an inhibitory site, ref. 29) was significantly increased (Figure 2, G and H). Given that urine osmolality was significantly lower in this group compared with vehicle control (Figure 2D), these findings suggest that the distal nephron was insensitive to vasopressin.

Renal prostaglandin E_2_ (PGE), endothelin-1 (ET-1), and NO play critical roles in fluid-electrolyte balance as paracrine/autocrine factors (23, 30, 31). Compared with vehicle-treated rats, MS275 rats had similar levels of PGE metabolite excretion in both the HSD and HSW rats (Supplemental Tables 2 and 3). ET-1 excretion was significantly increased in both HSD and HSW MS275-treated rats on day 7 compared with vehicle-treated rats (Supplemental Tables 2 and 3). Urinary NOx (nitrite + nitrate, metabolites of NO) excretion is a marker for renal NO production (23) and a proposed biomarker for the development of hypertension in humans (32). Urinary NOx excretion was significantly blunted in MS275-treated HSD (Figure 2I) and HSW rats (Figure 2J). Moreover, in the HSW rats, there was a significant decrease in urinary NOx excretion after only 2 days of MS275 treatment, suggesting that inhibition of class I HDACs attenuated the HSW-induced increase in urinary NOx (Figure 2J).

### Intramedullary HDAC inhibition reduces renal NO via decreased NO synthase expression.

One potential mechanism of reduced NOx excretion is that MS275 treatment results in an increase in reactive oxygen species (ROS). Urinary hydrogen peroxide excretion was similar between vehicle- and MS275-treated HSD rats (Supplemental Table 2). In contrast, urinary hydrogen peroxide excretion was significantly higher in HSW rats receiving MS275 (Supplemental Table 3). These data suggest that the method of increased salt consumption (eating versus drinking) while on HDACi can lead to differences in renal ROS production.

A second potential mechanism that reduces NO production involves changes in NO synthase (NOS) expression and/or activity. The inner medulla has the highest total NOS activity in the kidney and expresses all 3 NOS isoforms (22). There was a significant reduction in NOS1 (referred to as neuronal NOS or nNOS), both NOS1α and NOS1β splice variants, and NOS3 (endothelial NOS or eNOS) in the inner medulla of rats that received MS275 (Figure 2K). NOS2 (inducible NOS or iNOS) IM expression was not statistically different (Figure 2K). NOS3 activity is regulated through a number of posttranslational modifications. NOS3 phosphorylation of S1177, an activating site, was significantly reduced with MS275 treatment. However, this was driven by a decrease in total NOS3 expression (Supplemental Figure 3). MS275 treatment did not have a significant effect on the inhibitory phosphorylation site of NOS3 at T495 (Supplemental Figure 3).

### Collecting duct knockdown of Hdac1 reduces plasma potassium in males.

In the developing ureteric bud, *Hdac1* and *Hdac2* are essential for proper kidney development (26), but their role in the adult nephron remains elusive. To determine which kidney tubular cell types in adulthood may be significantly affected by HDAC inhibition, we generated 3 new inducible *Hdac1*- or *Hdac2*-knockdown murine models. First, *Hdac1* was genetically knocked down from the collecting duct in adulthood with doxycycline-inducible *Hdac1^fl/fl^* *Hoxb7-Cre* (i*Hoxb7 Hdac1KO*, Supplemental Figure 4). i*Hoxb7 Hdac1KO* mice had similar blood pressure to littermate control mice on an HSD as adults (Supplemental Table 1). When challenged with an HSD, i*Hoxb7 Hdac1KO* mice had a similar increase in natriuresis and diuresis as control mice (both sexes, Supplemental Table 5). They also presented with similar urinary NOx excretion (Supplemental Table 5). Although plasma sodium, chloride, and blood urea nitrogen (BUN) were similar between control and i*Hoxb7 Hdac1KO* of both sexes, plasma potassium was significantly lower in the male i*Hoxb7 Hdac1KO* mice (Supplemental Table 6).

### Collecting duct knockdown of Hdac1 and Hdac2 results in significant kidney injury, polyuria, and NO deficiency.

**Figure 3:**
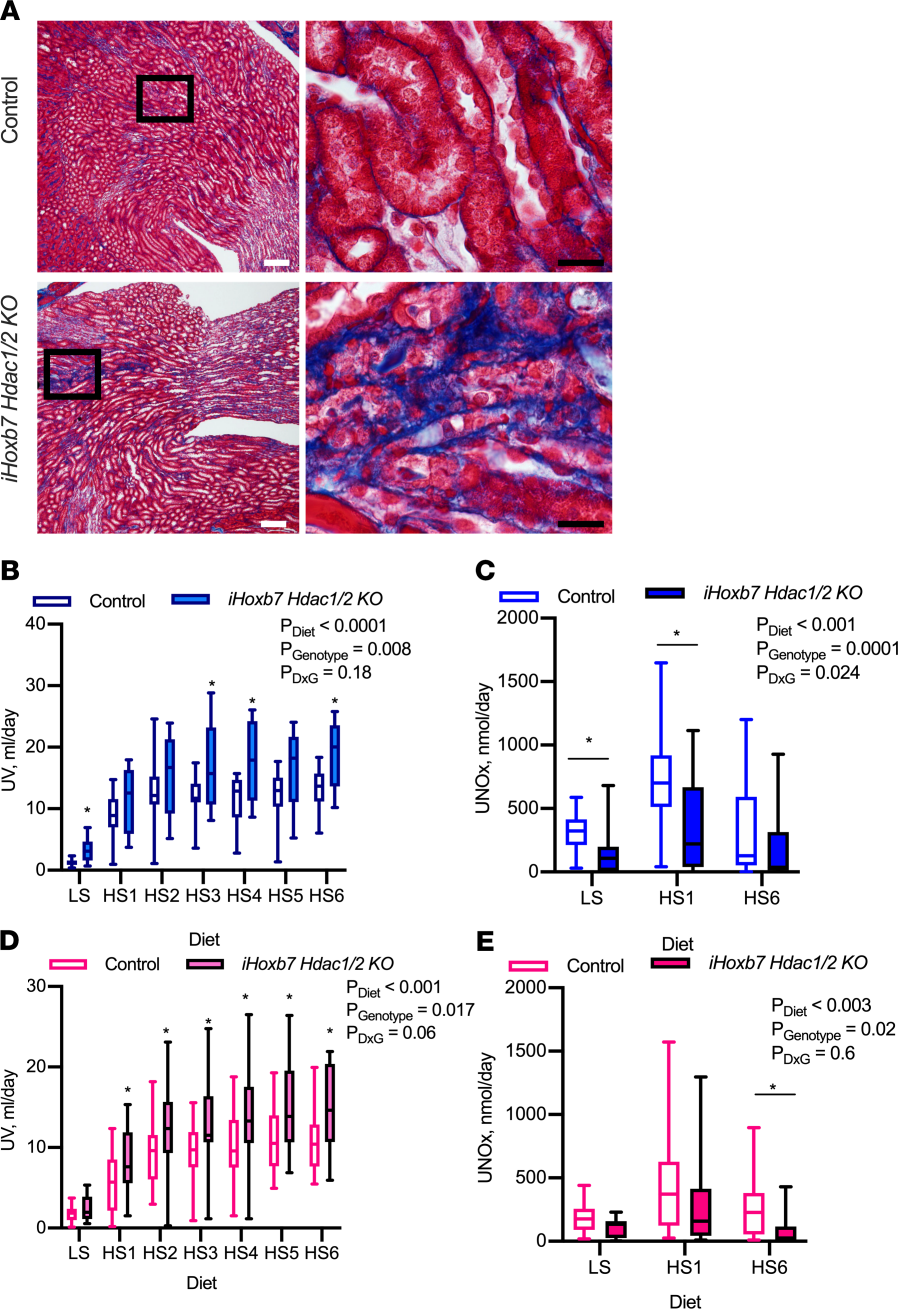
Collecting duct–specific deletion of *Hdac1* and *Hdac2* results in kidney damage and polyuria. (**A**) Representative Gomori’s trichrome staining images in control and *iHoxb7-rtTa-Lc-1-Cre Hdac1–*and *Hdac2*–knockout (*iHoxb7Hdac1/2KO*) mice (male control and KO *n* = 18 each, female control *n* = 18, KO = 12). KO mice present with significant interstitial fibrosis in areas with atrophied tubules. This was not observed in control mice. White scale bar: 100 μm. Black scale bar: 20 μm. (**B**) *iHoxb7Hdac1/2KO* male mice present with significant polyuria on a low-salt (LS) diet, which is further exacerbated on an HSD (HS). Box plots with median and maximum and minimum values plotted. (**C**) Male *iHoxb7Hdac1/2KO* mice have significantly lower urinary nitrite/nitrate (NOx) excretion on all diets. Individual data points plotted with mean ± SEM indicated. (**D**) Female *iHoxb7Hdac1/2KO* mice present with significant polyuria while eating an HSD, and this is associated with (**E**) lower urinary NOx excretion. Male control and KO *n* = 18 each, female control *N* = 18, KO = 12. Repeated-measures, 2-way ANOVA provided; asterisk represents significant difference from control as detected by post hoc Holm-Šidák test.

*Hdac1* and *Hdac2* were genetically knocked down from the collecting duct with a doxycycline-inducible *Hdac1^fl/fl^* *Hdac2^fl/fl^* *Hoxb7-Cre* (i*Hoxb7 Hdac1/2KO*, Supplemental Figure 5). Reduction of HDAC1 and HDAC2 in adulthood (Supplemental Figure 5) did not result in any mortality by 25 weeks of age. However, gross kidney abnormalities (hydronephrotic kidney, atrophied kidneys, and uninephrectomy) were apparent in 5/18 male i*Hoxb7 Hdac1/2KO* and 6/12 female i*Hoxb7 Hdac1/2KO*; none of the control male or female mice had gross kidney abnormalities. Damage was apparent, histologically presenting as dilated tubules, atrophied tubules, interstitial fibrosis, and protein casts, which were evident in 15/18 male and 11/12 female i*Hoxb7 Hdac1/2KO* kidneys (Figure 3A). For controls 1/20 males and 3/18 females had mild damage with 2 or fewer protein casts detected. At 13 ± 4 weeks of age, mice were fed a low-sodium diet (<0.01% NaCl) followed by a week of HSD (4.0% NaCl) following our previously published protocols (23, 24). Urine flow was significantly higher in male i*Hoxb7 Hdac1/2KO* mice compared with littermate controls on all salt diets (Figure 3B, Supplemental Table 7), but sodium excretion was similar between the genotypes (Supplemental Table 7). Urinary NOx excretion was significantly attenuated on all salt diets in the male i*Hoxb7 Hdac1/2KO* mice (Figure 3C). Female i*Hoxb7 Hdac1/2KO* mice presented with significant polyuria on an HSD compared with littermate control female mice (Figure 3D, Supplemental Table 7) but had similar natriuresis to controls (Supplemental Table 7). The female knockout mice also presented with significantly lower urinary NOx excretion on all salt diets compared with controls (Figure 3E). Plasma electrolytes were similar between female control and i*Hoxb7 Hdac1/2KO*, but the KO mice had an elevated BUN (Supplemental Table 8) consistent with the kidney damage observed. Male i*Hoxb7 Hdac1/2KO* mice also had a mild, but statistically significant, decrease in plasma Na compared with controls on both low-salt diets and HSDs (Supplemental Table 8). Plasma BUN was elevated in the male i*Hoxb7 Hdac1/2KO* mice (Supplemental Table 8) consistent with the kidney damage observed (Figure 3). Blood pressure was similar among controls and i*Hoxb7 Hdac1/2KO* male and female mice on an HSD (Supplemental Table 1). Knockdown of *Hdac1* and *Hdac2* from the collecting duct resulted in high salt–mediated polyuria and kidney NO deficiency that was independent of blood pressure.

### Whole-nephron Hdac1 and Hdac2 knockdown results in kidney damage, plasma electrolyte imbalance, and death.

**Figure 4:**
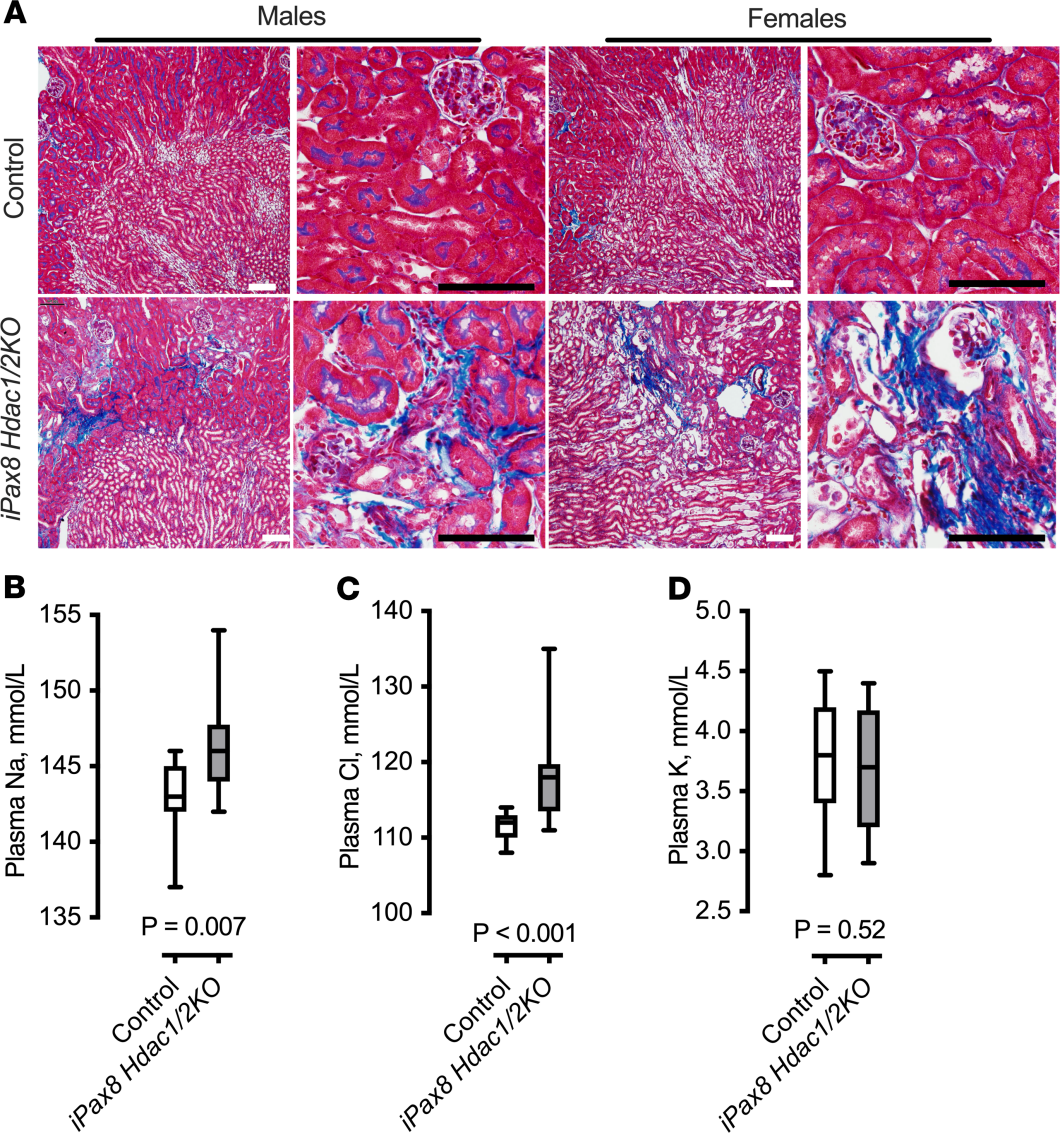
i*Pax8 Hdac1/2KO* mice have significant kidney damage. (**A**) Representative images of Gomori’s trichrome staining in control and i*Pax8 Hdac1/2KO* mice (from a total of *n* = 8 per sex, per group). KO mice present with interstitial fibrosis in areas with atrophied tubules in both male and female mice. This was not observed in control mice. White scale bar: 100 μm. Black scale bar: 20 μm. (**B**–**D**) The *iPax8 Hdac1/2KO* mice (male and female data combined) present with (**B**) higher plasma sodium and (**C**) plasma chloride but (**D**) normal plasma potassium. Control *n* = 23, KO = 16. Box plots show median and maximum and minimum values plotted. Results of unpaired, 2-tailed Student’s *t* test are in each panel.

Using doxycycline-inducible *Pax8-rtTA/Lc-1* (33, 34), *Hdac1* and *Hdac2* were knocked down from the kidney epithelium (i*Pax8-rtTA/Lc-1* *Hdac1/2KO*, Supplemental Figure 6, A and B). Samples were collected 2 weeks after doxycycline, and i*Pax8 Hdac1/2KO* male and female mice presented with significantly higher kidney/body mass ratios (Supplemental Figure 6C) and substantial interstitial fibrosis and tubular injury (Figure 4A). We next determined the effect of kidney epithelial knockdown of *Hdac1* and *Hdac2* on plasma electrolytes. Plasma electrolyte measurements in the i*Pax8 Hdac1/2KO* mice presented with significantly higher plasma sodium (Figure 4B and Supplemental Figure 7A), and chloride (Figure 4C and Supplemental Figure 7B), but similar levels of potassium (Figure 4D and Supplemental Figure 7C). In both adult male and female knockout mice (mean age 14 ± 2 weeks), 27–28 days after doxycycline 100% mortality occurred (*n* = 6); none of the control mice died (*n* = 6).

### Kidney epithelial Hdac1 and Hdac2 regulate ion and water transporter transcriptomics.

**Figure 5:**
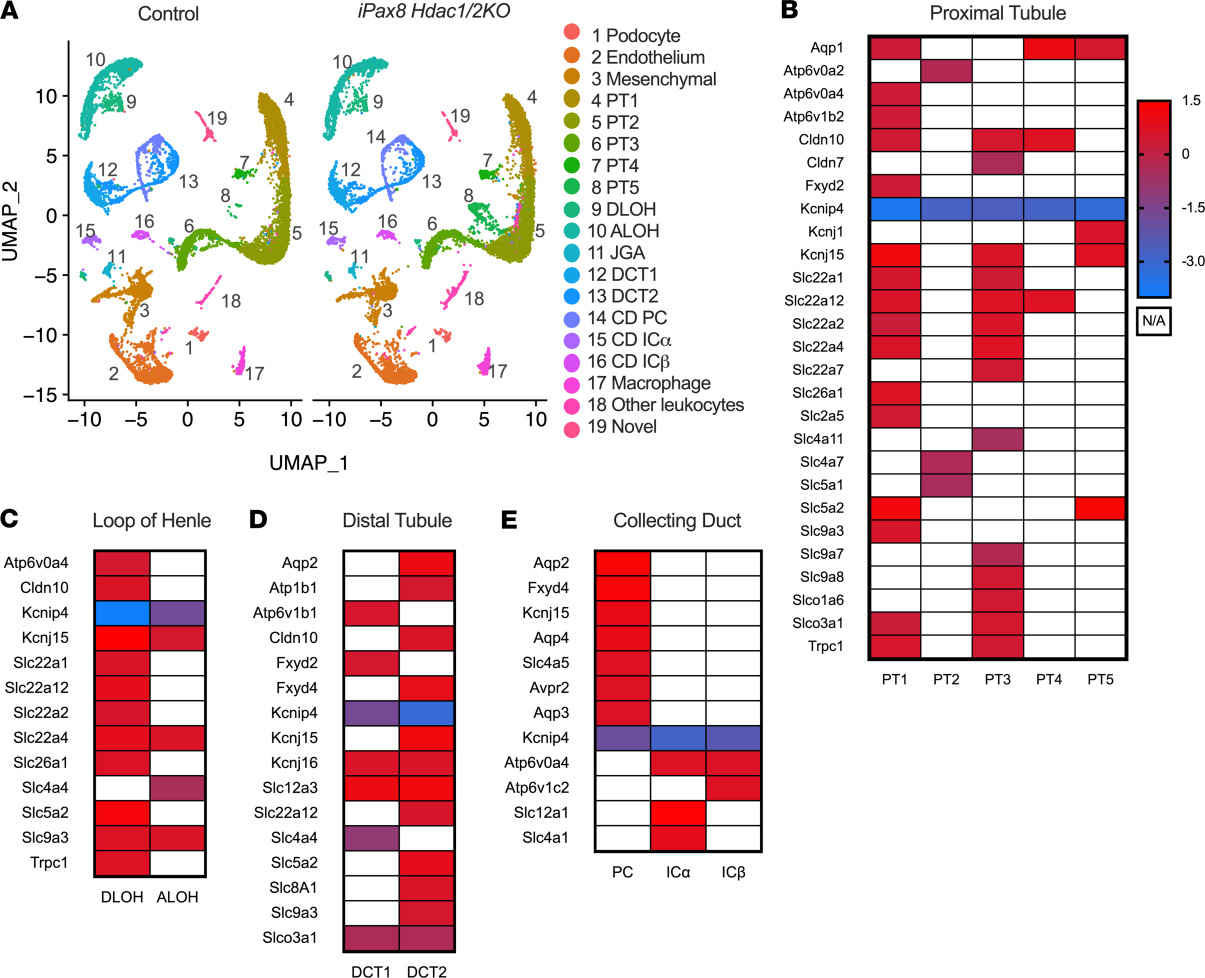
Integrated data set of snRNA-Seq results of control and i*Pax8 Hdac1/2KO* male and female mice. (**A**) Cluster 8 is abundant in the knockout. (**B**–**E**) Control mice as compared with i*Pax8 Hdac1/2KO* mice have significantly higher expression of genes involved in fluid-electrolyte balance. (**B**) Differentially expressed genes in the proximal tubular cells, (**C**) loop of Henle, (**D**) distal tubules, and (**E**) collecting ducts. Heatmap legend represents log fold change (control/i*Pax8 Hdac1/2KO*), and white represents not available (N/A, not significantly expressed). PT, proximal tubule; DLOH, descending loop of Henle; ALOH, ascending loop of Henle; DCT, distal tubules; JGA, juxtaglomerular apparatus; CD, collecting duct; PC, principal cell; ICα, intercalated cell type A; ICβ, intercalated cell type B.

Determinations of the effect of kidney epithelial knockdown of *Hdac1* and *Hdac2* on individual cell types in the kidney were conducted with single-nucleus RNA-sequencing (snRNA-Seq). From male and female control and i*Pax8 Hdac1/2KO* mice, 25,075 nuclei were sequenced, and 19 clusters of kidney cells were identified (Figure 5A, Supplemental Figure 8, and Supplemental Table 9). Within each cluster, the differentially expressed genes between control and i*Pax8 Hdac1/2KO* were determined (Supplemental Figures 9–14 and Supplemental Table 10). Within a cluster, the number of genes up- or downregulated with *Hdac1/Hdac2* KO was similar, suggesting HDAC1 and HDAC2 both promote and inhibit transcription (Supplemental Figures 9–14). Consistently across cell clusters, ion channels, ion transporters, and water channels were significantly higher in control mice compared with KO. This included proximal tubular cell (PTC) sodium glucose co-transporter-1 (SGLT1, *Slc5a1*) and -2 (SGLT2, *Slc5a2*, Figure 5B), sodium/hydrogen exchanger-3 (NHE3, *Slc9a3*) in the loop of Henle (Figure 5C), sodium/chloride cotransporter (NCC, *Slc12a3*) in the distal tubule (Figure 5D), and AQP2 and -3 in the collecting duct (*Aqp2*, *Aqp3*, Figure 5E). Thus, in the adult nephron, HDAC1 and HDAC2 are necessary for kidney health and maintaining proper plasma electrolyte concentrations.

**Figure 6:**
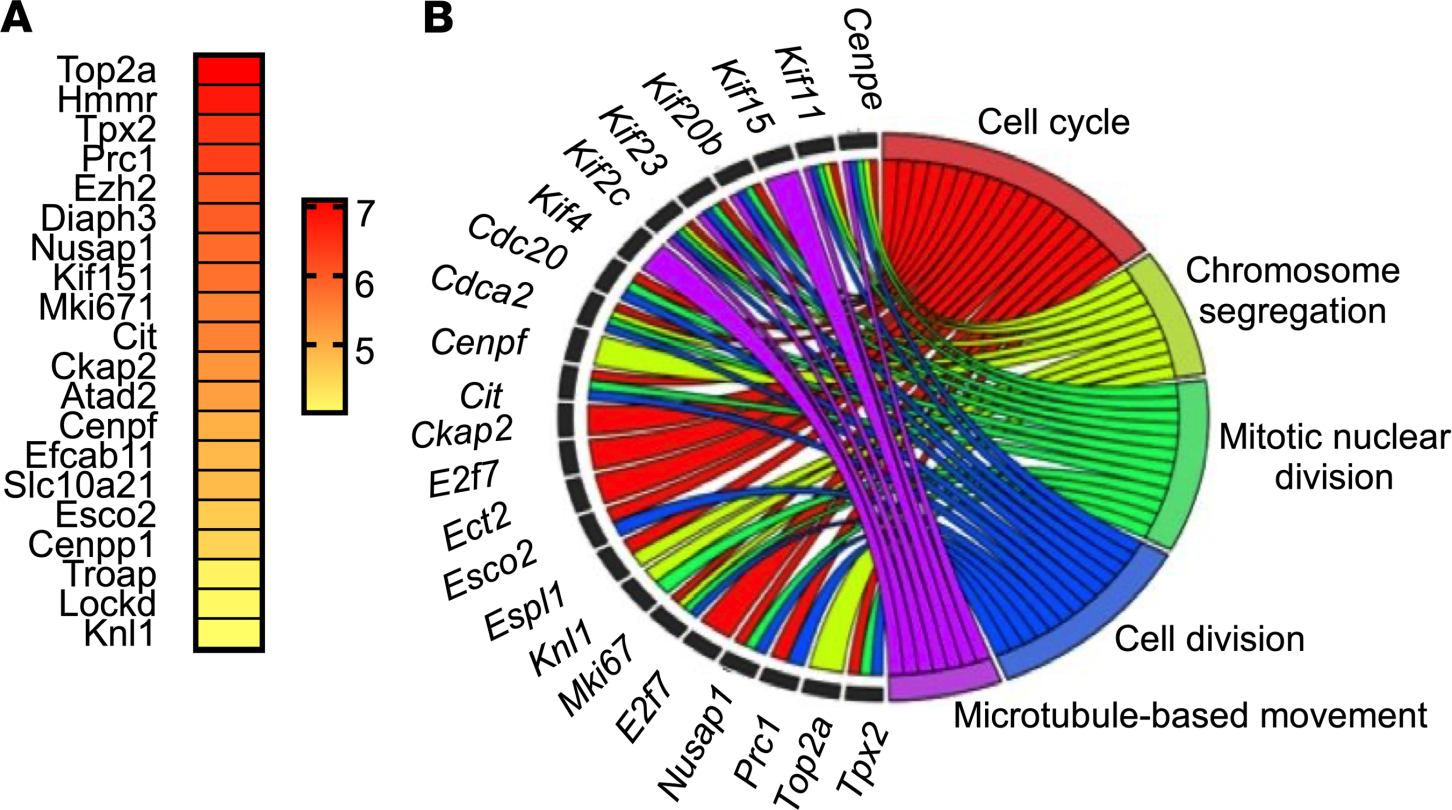
i*Pax8 Hdac1/2KO* mice have a unique cluster of kidney cells, cluster 8. (**A**) A heatmap of the top 20 highly differentially expressed gene markers in cluster 8. (**B**) Summary of the significant Gene Ontology biological processes in cluster 8 compared with all other clusters and relationships among the genes in these pathways.

These data also revealed a unique cluster of PTC (cluster 8, named PT5) that was highly expressed in the KO (Figure 5A), and the highest differentially expressed gene in this cluster was DNA topoisomerase II alpha (*Top2a*) (Figure 6A). Gene Ontology analysis of cluster 8 determined this cluster is significantly enriched with genes associated with the biological pathways: cell cycle, chromosomal segregation, mitotic nuclear division, cell division, and microtubule-based movement (Figure 6B). Thus, epithelial *Hdac1* and *Hdac2* deletion results in a novel population of PTC that has altered mitosis consistent with the histological results.

### Meta-analysis of fluid-electrolyte disorders with HDACi.

**Figure 7:**
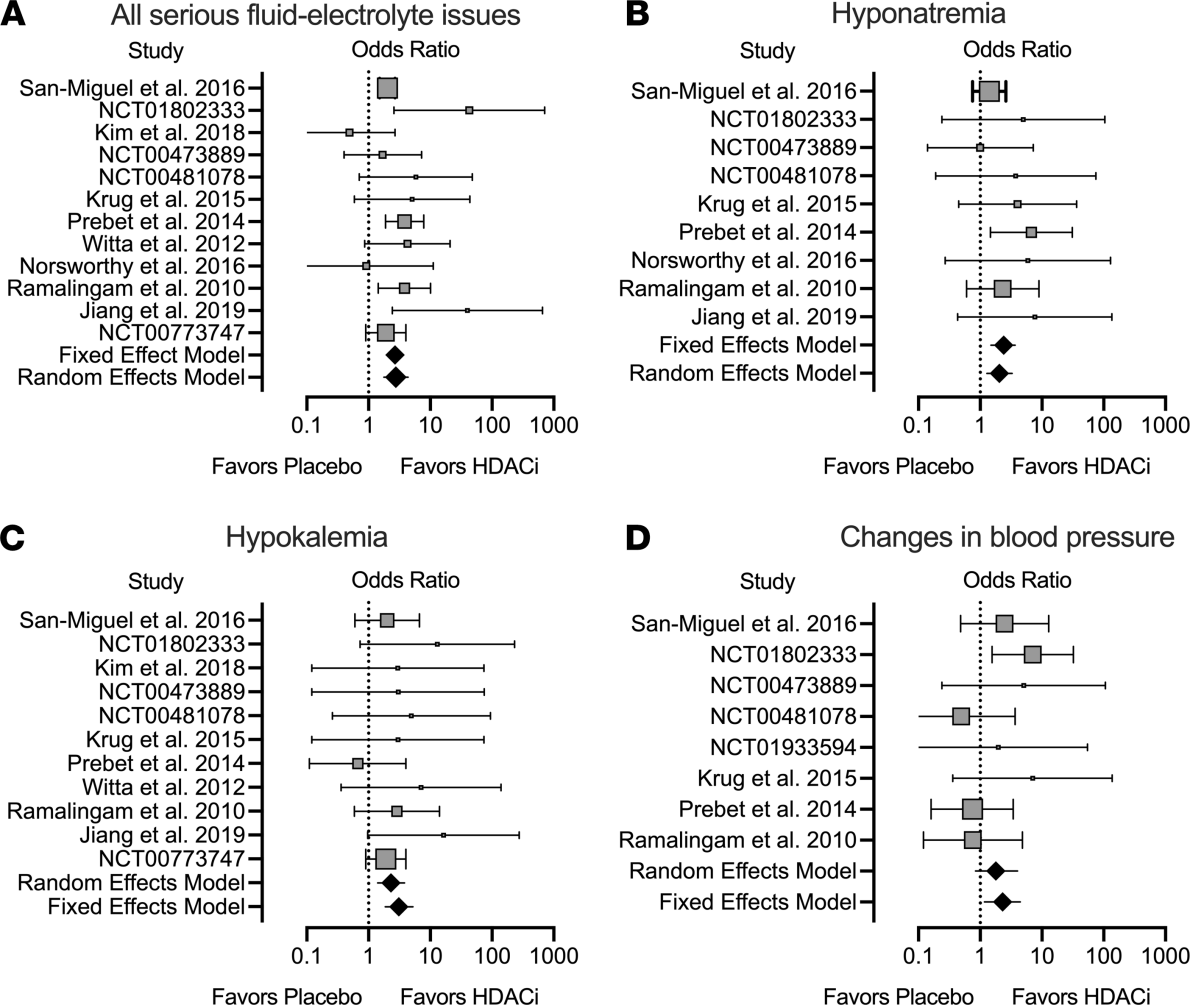
Meta-analysis of serious adverse (≥ grade 3) fluid-electrolyte abnormalities and blood pressure in subjects on placebo or standard therapy versus those on HDACi. Forest plots of the OR with horizontal lines representing 95% CI and diamonds representing OR for all studies combined. There is a significant increase in odds of (**A**) any fluid-electrolyte abnormality (*P* < 0.0001), (**B**) hyponatremia (*P* = 0.0003) or (**C**) hypokalemia (*P* < 0.0001) or (**D**) a change in blood pressure (either increase or decrease, *P* = 0.015) with HDACi use.

A systematic review with a meta-analysis was conducted with data from the literature and clinical trials. Only studies that included serious adverse events (defined as grade ≥ 3) for both placebo/standard of care versus HDACi were included (Supplemental Figure 15). Subjects receiving an HDACi had a significantly greater OR of having a serious fluid-electrolyte disorder (OR = 2.7 [95% CI 2.2 to 3.4], *P* < 0.0001) (Figure 7A and Supplemental Figure 16A). This includes a significant OR of 2.4 (95% CI 1.5 to 3.8, *P* = 0.0003) for hyponatremia (Figure 7B and Supplemental Figure 16B) or OR 3.1 (95% CI 1.2 to 4.4, *P* < 0.0001) for hypokalemia (Figure 7C and Supplemental Figure 17A). A significant change in blood pressure was also associated with use of HDACi (OR 2.28 [95% CI 1.2 to 4.4], *P* = 0.015; Figure 7D and Supplemental Figure 17B). Use of HDACi is associated with a significant increase in risk of severe fluid-electrolyte disorders in human subjects.

## Discussion

Cardiovascular and renal diseases are the leading causes of death worldwide; thus, there is a critical need to identify mechanisms and to develop novel therapies. The main finding from this study reveals that kidney epithelial HDACs are critical in regulating fluid-electrolyte balance. The data indicate that class I HDACs (HDAC1 and HDAC2) specifically influence transcription and protein abundance/activity of ion transporters and ion or water channels in the kidney epithelium. Moreover, class I HDACs are necessary for high salt–mediated activation of the NOS/NO pathway. This study highlights the potential risk of HDACi on fluid-electrolyte disorders.

Evidence from models of heart failure (13), ischemia/reperfusion injury (14), and diabetic nephropathy (15) have suggested that HDACi use may prevent fibrosis and inflammation in these diseases, appearing to be a promising therapeutic approach. Clinical data suggest that HDACi use leads to loss of homeostatic mechanisms in fluid-electrolyte balance because reported side effects include hyponatremia, hypokalemia, edema, and changes in blood pressure. These adverse events are found in a majority of the HDACi clinical trials registered with ClinicalTrials.gov, demonstrating a common and significant problem that may be fatal for patients with cardiovascular or kidney disease. Indeed, the meta-analysis presented in this study demonstrates that there is a significant increased risk of fluid-electrolyte disorders in subjects using HDACi.

Class I *Hdacs* have relatively high mRNA expression in the distal nephron of the rat (25). We previously reported the localization of kidney HDACs in mice (27), and here we present that in both humans and rats, HDAC1, HDAC2, HDAC3, and HDAC8 proteins are present in the kidney. Moreover, IM HDAC1 and to a lesser extent HDAC3 are increased in response to a chronic HSD. These data suggest that class I HDACs have a physiological role in the kidney and may be involved in regulating homeostatic mechanisms of fluid-electrolyte balance.

To define the role of class I HDACs in the adult kidney, multiple approaches and models were used to model human health. First, kidney medullary class I HDACs were inhibited in salt-loaded rats. Class I HDAC inhibition with MS275 (entinostat), which is currently used in clinical trials for cancer (35), resulted in polyuria, kidney NO deficiency, and marked increase in mean arterial pressure within 7 days of salt loading. Similarly, in mice where collecting duct HDAC1 and HDAC2 insufficiency was induced in adulthood, there were polyuria, NO deficiency, and kidney damage, independent of blood pressure. From the clinical data, urine incontinence (the need to frequently void), increased urine output, and thirst were documented in subjects treated with HDACi (36, 37) (NCT01802333, NCT00481078). The polyuria with HDACi is likely derived from kidney dysfunction because in response to renal intramedullary infusion of HDACi, collecting duct AQP2 (the vasopressin-sensitive apical water channel that is required for concentrating urine) was markedly downregulated and phosphorylated at an inhibitory site. From our single-nucleus transcriptome, HDAC1 and HDAC2 in the principal cell are necessary for proper *Aqp2* and *Aqp3* transcription. Thus, HDACs play a novel role in the regulation of kidney aquaporin transcription and abundance.

Dysnatremia and dyskalemia (either hyper- or hypo-natremia/kalemia) are common and serious electrolyte disturbances (38, 39). For example, a retrospective study of emergency room admissions determined that electrolyte imbalances are significantly associated with 30-day and 1-year mortality (39). Likewise, as many as 20% of hospitalized patients have serious complications from hyponatremia (20) and hypokalemia (40), and both are associated with an increase in mortality. Thus, use of HDACi is very significant to human health and leads to an OR greater than 2 for a fluid-electrolyte disorder. All rats with HDACi and nephron or collecting duct–specific *Hdac1/Hdac2* knockdown models presented with fluid-electrolyte imbalances. The whole-nephron HDAC1/HDAC2-deficient animals had the most severe imbalances, presenting with significant hypernatremia/hyperchloremia and death within 30 days of knockdown. Single-nucleus transcriptomics also highlighted that epithelial HDAC1 and HDAC2 are necessary for the expression of ion transporters and channels. In each kidney cell cluster, numerous solute carriers were significantly affected, including those critical for sodium retention: SGLT2, NHE3, and NCC (Figure 5). Thus, kidney epithelial HDAC1 and/or HDAC2 are critical for fluid-electrolyte balance.

Kidney epithelial HDAC1 and HDAC2 are also critically important for maintenance of a healthy tubulointerstitium. Kidney injury marker-1 (gene *Havcr1*), a PTC injury marker (41), was increased in the PTC cluster 6 of the knockout mice. In many cell clusters, glutathione peroxidase-3 (*Gpx3*) was markedly decreased in the whole-nephron HDAC1/HDAC2-KO mice. Gpx3 is synthesized in the kidney, and it functions systemically to reduce ROS (42). Patients with chronic kidney disease (CKD) are deficient in GPX3 (43); moreover, preclinical studies determined that GPX3 deficiency is a significant risk factor for cardiovascular disease in CKD (44). Kidney epithelia lacking *Hdac1/Hdac2* also have reduced acyl-coenzyme A synthetase (ACSM2), a gene involved in fatty acid metabolism in adulthood. This deficiency in ACSM2 was observed in all kidney cells (epithelial, endothelial, mesenchymal, and immune). ACSM2 was reported as significantly reduced in the developing kidney lacking HDAC1/HDAC2 in nephron progenitor cells (45). *Acsm2* transcript abundance in the kidney is positively correlated with estimated glomerular filtration rate in human subjects (46). These data suggest that HDAC1 and HDAC2 substantially affect fatty acid metabolism and kidney function. We also observed histological evidence of kidney damage in the whole-nephron *Hdac1/Hdac2KO* and collecting duct *Hdac1/Hdac2KO* mice. Hydronephrosis was prevalent (30%) in male and female collecting duct *Hdac1/Hdac2KO* mice. Chronic excess urination can lead to bladder distention, renal back pressure leading to kidney atrophy, or even hydronephrosis in humans (47, 48). Thus, these data indicate that epithelial HDAC1 and HDAC2 are necessary to prevent excess urination in both males and females and promote a healthy tubulointerstitium.

In the whole-nephron *Hdac1/Hdac2KO* mice, there were similar percentages of genes expressed higher or lower compared with control, suggesting HDAC1 and HDAC2 both promote and silence transcription. This was also found in the developing ureteric bud, where HDAC1/HDAC2 knockdown resulted in 226 genes increased and 270 genes decreased (out of ~41,000 probes) (26). Adult whole-nephron *Hdac1/Hdac2* knockdown mice of both sexes had a unique PTC cluster that expressed *Top2a* 128-fold higher than all other kidney cell types. Top2a is expressed during G2/M phase and functions in generating DNA breaks and ligation needed for chromosome separation during mitosis. HDAC1 and HDAC2 directly interact with Top2a and are functionally coupled in the nucleus (49, 50). Excessive TOP2A leads to uncontrolled proliferation; as such, combined HDACi and topoisomerase inhibitors are hypothesized to induce apoptosis in cancer (51). These data suggest that unique populations of PTCs with HDAC1/HDAC2 deletion are proliferating cells. Frequently among the cluster transcriptomes, ectodysplasin-A (*Eda*) was substantially increased in the KO mice. EDA is a cytokine and part of the tumor necrosis factor family that functions in ectodermal organ development. In adulthood it is expressed in the kidney, and it functions to promote epithelial barrier function (52). EDA was reported to be significantly lower in peripheral blood mononuclear cells of CKD and end-stage renal disease subjects (53). The functional consequence of a significant increase in kidney EDA warrants further investigation; however, increased EDA was also observed in the developing kidney of HDAC1/HDAC2 nephron progenitor cell mice (45). A limitation to our transcriptome analysis is that the direct targets of HDAC1 and HDAC2 cannot be distinguished from indirect targets. Furthermore, it is evident that knockdown of kidney epithelial HDAC1/HDAC2 in adulthood results in significant transcriptional changes in all cell clusters of the kidney and not exclusively limited to the tubular structures, indicating that HDAC1 and HDAC2 also regulates aspects of cellular crosstalk or potential paracrine mediators, such as NO.

Chronic blood pressure control is maintained through the regulation of extracellular fluid volume. There is a complex interaction among antinatriuretic and natriuretic neurohumoral, paracrine, and autocrine factors in order to keep blood pressure within the normal set point. Although dysregulated HDAC activity may lead to hypertension and data suggest that in angiotensin II– or obesity-induced hypertension HDACi may lower pressure (54–56), we also found in our meta-analysis that HDACi led to a significant risk of a change in pressure. In the HDACi clinical trials, both hypertension and hypotension were reported, and the effect of HDACi on a change in pressure among individual trials was variable (Figure 7). HDACi led to increased blood pressure in salt-loaded rats but not mice in our study, the reason(s) for which requires further investigation. Yet, our study highlights an important area of study because more HDACi are being approved to treat additional cancers, and consequently patient exposure to HDACi will increase. Monitoring blood pressure will be essential to prevent serious events related to hypotension or hypertension with exposure to HDACi.

We report data that HDACi led to significant changes in the kidney NO system. Urinary NOx excretion reflects both dietary nitrite/nitrate excretion and renal NO production (23). HDACi or *Hdac1/Hdac2* deletion resulted in reduced urinary NOx excretion. These data suggest that HDACs are novel regulators that promote NO production during high-salt feeding. In agreement with this was the discovery that treatment with HDACi resulted in a significant decrease in IM NOS1 and NOS3 expression (Figure 2K). These data suggest that class I HDACs promote NOS1 and NOS3 abundance during chronic high-salt intake in rats. This finding is clinically relevant because there are associations between reduced NO and hypertension, and even salt-sensitive hypertension, in humans (57–59), rats (60), and mice (23). Moreover, patients with kidney disease often present with a salt-sensitive blood pressure. In a study with type 2 diabetic nephropathy subjects compared with type 2 diabetics without kidney disease, urinary NOx was blunted and failed to increase with HSD feeding, and this was associated with a salt-sensitive rise in mean arterial pressure (61). Even chronic kidney disease patients, adult and pediatric, have reduced NO, and this is associated with increased cardiovascular disease risk (62, 63). A recent pilot study with CKD subjects determined that increasing NO in the body, by an acute dietary nitrate load (which is reduced to NO in the body), significantly reduced blood pressure and renal resistive index (64). Thus, kidney HDAC1 and HDAC2 play a novel function in the regulation of kidney NO status, likely through modulating NOS abundance. This suggests that chronic use of HDACi may exacerbate NO deficiency and increase cardiovascular disease risk in patients with kidney disease.

To conclude, we present new evidence that renal medullary class I HDACs play a critical role in the regulation of homeostatic mechanisms involved in maintaining fluid-electrolyte balance. Kidney class I HDACs, especially HDAC1, are increased with dietary sodium, and it is well appreciated that a Western diet is high in sodium. Thus, kidney HDACs provide a link between the environment, such as consumption of HSDs, with regulation of homeostatic mechanisms to remain in fluid-electrolyte balance. Given the research of HDACi to treat cancers, cardiovascular diseases, and kidney diseases, this study sheds light on the mechanism(s) of the adverse events in fluid-electrolyte homeostasis that may be fatal to critically ill patients.

## Methods

### Rats, telemetry, and chronic intramedullary infusion.

Sprague-Dawley, 8-week-old, male rats (225 g) were purchased from Harlan (now Envigo) and maintained on a 12-hour light/12-hour dark schedule. These rats were fed a normal-salt diet (0.49% NaCl, Teklad 96208) and water ad libitum. At 10 weeks of age, rats (*N* = 5) were randomly assigned to either normal-salt diet or HSD (4.0% NaCl, Teklad 92034) for 7 days. These rats were then euthanized and plasma, kidney cortex, outer medulla, and inner medulla were dissected and snap-frozen for Western blot experiments.

At 9 weeks of age, male Sprague-Dawley rats underwent uninephrectomy leaving the right kidney intact and were implanted with telemetry devices (Data Sciences Inc) as previously described (65). After a week of recovery, iPRECIO microinfusion pumps (SMP-200) were implanted into the right kidney as previously described (66). The pumps were programmed to deliver at a rate of 9 μL/min for the duration of the study. This rate of delivery was based on previous studies that determined 10 μL/min infusion of 0.9% saline had no significant effect on renal hemodynamics (67). Details of the surgery are found in the Supplemental Methods.

### Infusion protocol 1, feeding of HSD and access to free water.

Rats were placed in metabolic cages with 4.0% NaCl powdered diet (Teklad 92034) and had free access to water (HSD). The pumps were filled with vehicle (30% DMSO in 0.9% saline, all solutions sterile, *N* = 4) or the class I HDAC inhibitor MS275 (68) (Cayman Chemical, *N* = 5), at 1 mg/kg/d. This concentration was used based on pharmacokinetic studies in rats that gave oral doses of 15 mg/kg (69).

### Infusion protocol 2, 1% NaCl in water and normal-salt diet.

Rats were placed on 1% NaCl drinking water and maintained on 0.49% NaCl ground diet (Teklad 96208) (HSW, *N* = 5). The iPRECIO pumps were filled with vehicle (30% DMSO in 0.9% saline, all solutions sterile) for 7 days. Following this, the pumps were refilled with MS275 (1 mg/kg/d) for an additional 7 days according to the manufacturer’s directions.

### Murine models.

All details of the genetics, genotyping, inducible knockdown strategies, and confirmation of knockdown are provided in the Supplemental Methods. *Hdac1^fl/fl^* and *Hdac2^fl/fl^* mice were a gift from Eric Olson (University of Texas Southwestern, Dallas, Texas, USA). To generate inducible kidney epithelial *Hdac1*- and *Hdac2*-KO mice, HDAC1*^fl/fl^* and HDAC2*^fl/fl^* mice were bred with doxycycline-inducible *Pax8*-reverse tetracycline transactivator (rtTA) (34) and the bicistronic Cre (LC-1) (33) hemizygous mice. Upon doxycycline treatment of males and females, mice with the genotype *Hdac1^fl/fl^* *Hdac2^fl/fl^* *Pax8-rtTA-Lc-1* would be KO animals, and littermate controls had the following possible genotypes: (a) *Hdac1^fl/fl^* and *Hdac2^fl/fl^*, (b) *Hdac1^fl/fl^* *Hdac2^fl/fl^* *Pax8-rtTA*, (c) *Hdac1^fl/fl^*, and (d) *Hdac2^fl/fl^* *Lc-1*. Collecting duct–specific HDAC1- and HDAC2-KO mice were generated by breeding *Hdac1^fl/fl^* and *Hdac2^fl/fl^* with doxycycline-inducible *Hoxb7-rtTA-Lc-1* mice (The Jackson Laboratory stock 016567).

Floxed HDAC2 alleles were bred out by mating *Hdac1^fl/fl^* and *Hdac2^fl/fl^* mice with a C57blk/6J mice and then bred back to only *Hdac1^fl/fl^*. Collecting duct *Hdac1*-KO and littermate control mice were generated by breeding *Hdac1^fl/fl^* with *Hoxb7-rtTA Lc-1* mice. All mice in these models were provided doxycycline at 6–8 weeks of age to induce KO in the *rtTA/Lc-1*–positive mice. Knockdown was confirmed by PCR (Supplemental Table 11 for primers) for the recombinant alleles of *Hdac1* and/or *Hdac2*, and immunolocalization studies are described in the online Supplemental Methods.

### Murine salt loading, metabolic cages, and telemetry.

Mice were provided gel diets with different amounts of NaCl as described in detail elsewhere (24) for both the metabolic cage and telemetry studies. Mice were individually housed in the metabolic cages as previously described (24). Following the metabolic cage study, mice underwent telemetry surgery as previously described (23).

### Urine and plasma analyses and Western blots.

See Supplemental Methods and Supplemental Table 12 for antibodies used in the study.

### Single-nucleus RNA-sequencing.

Nuclei suspensions were generated from male and female control and i*Pax8 Hdac1/2KO* mice following the methods of Wu et al. (70). The 10x Genomics platform was used to analyze the transcriptome of single nuclei, and raw and processed files were deposited in the National Center for Biotechnology Information’s Gene Expression Omnibus (GSE148354). Precise methods and details of data analyses are found in the online supplemental materials.

### Systemic review and meta-analysis.

PubMed and ClinicalTrials.gov searches were performed in August of 2019 and are described in detail in the Supplemental Methods. Only serious adverse events of grade 3 or higher were included in the analysis. The Mantel-Haenszel method was used following the methods and code of Efthimiou (71). ORs and 95% CIs were calculated and analyzed using both fixed and random effects models. Forest plots were generated with the data set using R.

### Statistics.

Data are reported as either individual points with mean ± SEM plotted or represented by box plots with median marked and minimum to maximum values denoted by vertical lines. For dietary salt interventions, all data were analyzed using repeated-measures, 2-way ANOVA (time and drug or genotype and diet) with post hoc Holm-Šidák multiple comparison test. For the HSW study, 2-group comparisons were performed using a paired, 2-tailed Student’s *t* test to compare either day 7 of vehicle and day 7 of MS275 or the change from day 2 or 7 to day 1 of vehicle or MS275. For 2-mean comparisons a 2-tailed Student’s *t* test was used. α = 0.05 and *P* < 0.05 were considered statistically significant. Significance of results from the meta-analysis was determined by both fixed and random models.

### Study approval.

All animal use and welfare adhered to the NIH *Guide for the Care and Use of Laboratory Animals* (National Academies Press, 2011) following a protocol reviewed and approved by the Institutional Laboratory Animal Care and Use Committee of the University of Alabama at Birmingham. Human kidney lysate samples were purchased from OriGene. OriGene collected these samples from US institutions under strict IRB and ethical consent practices.

## Author contributions

KAH, JSS, DMP, and JSP designed research. KAH, JSS, LDM, JMA, JC, RS, CJ, and HJJ performed research. KAH, JSS, SED, DMP, and JSP provided resources for the research. KAH, JSS, HJJ, DMP, and JSP analyzed data. KAH and JSP wrote the paper. All authors approved the final manuscript.

## Supplementary Material

Supplemental data

Supplemental Table 9

Supplemental Table 10

## Version 1 07/16/2020

In-Press Preview

## Version 2 08/20/2020

Electronic publication

## References

[1] Hall JE (2012). Hypertension: physiology and pathophysiology. Compr Physiol.

[2] Qi H (2018). Effects of environmental and genetic risk factors for salt sensitivity on blood pressure in northern China: the systemic epidemiology of salt sensitivity (EpiSS) cohort study. BMJ Open.

[3] Leyvraz M (2018). Sodium intake and blood pressure in children and adolescents: a systematic review and meta-analysis of experimental and observational studies. Int J Epidemiol.

[4] Borah PK (2018). Salt-sensitive phenotypes: A community-based exploratory study from northeastern India. Natl Med J India.

[5] Weinberger MH, Fineberg NS, Fineberg SE, Weinberger M (2001). Salt sensitivity, pulse pressure, and death in normal and hypertensive humans. Hypertension.

[6] Gu Q, Burt VL, Dillon CF, Yoon S (2012). Trends in antihypertensive medication use and blood pressure control among United States adults with hypertension: the National Health And Nutrition Examination Survey, 2001 to 2010. Circulation.

[7] Hyndman KA (2020). Histone deacetylases in kidney physiology and acute kidney injury. Semin Nephrol.

[8] Mottamal M, Zheng S, Huang TL, Wang G (2015). Histone deacetylase inhibitors in clinical studies as templates for new anticancer agents. Molecules.

[9] Plumb JA (2003). Pharmacodynamic response and inhibition of growth of human tumor xenografts by the novel histone deacetylase inhibitor PXD101. Mol Cancer Ther.

[10] Fenichel MP (2015). FDA approves new agent for multiple myeloma. J Natl Cancer Inst.

[11] Furumai R (2002). FK228 (depsipeptide) as a natural prodrug that inhibits class I histone deacetylases. Cancer Res.

[12] Kim HJ, Bae SC (2011). Histone deacetylase inhibitors: molecular mechanisms of action and clinical trials as anti-cancer drugs. Am J Transl Res.

[13] McKinsey TA (2012). Therapeutic potential for HDAC inhibitors in the heart. Annu Rev Pharmacol Toxicol.

[14] Levine MH (2015). Class-specific histone/protein deacetylase inhibition protects against renal ischemia reperfusion injury and fibrosis formation. Am J Transplant.

[15] Advani A (2011). Long-term administration of the histone deacetylase inhibitor vorinostat attenuates renal injury in experimental diabetes through an endothelial nitric oxide synthase-dependent mechanism. Am J Pathol.

[16] Ree AH (2010). Vorinostat, a histone deacetylase inhibitor, combined with pelvic palliative radiotherapy for gastrointestinal carcinoma: the Pelvic Radiation and Vorinostat (PRAVO) phase 1 study. Lancet Oncol.

[17] Ramalingam SS (2010). Carboplatin and Paclitaxel in combination with either vorinostat or placebo for first-line therapy of advanced non-small-cell lung cancer. J Clin Oncol.

[18] Pili R (2012). Phase I study of the histone deacetylase inhibitor entinostat in combination with 13-cis retinoic acid in patients with solid tumours. Br J Cancer.

[19] Woyach JA (2009). Lack of therapeutic effect of the histone deacetylase inhibitor vorinostat in patients with metastatic radioiodine-refractory thyroid carcinoma. J Clin Endocrinol Metab.

[20] Rafat C, Flamant M, Gaudry S, Vidal-Petiot E, Ricard JD, Dreyfuss D (2015). Hyponatremia in the intensive care unit: how to avoid a Zugzwang situation?. Ann Intensive Care.

[21] Kitamura K, Tanaka T, Kato J, Ogawa T, Eto T, Tanaka K (1989). Immunoreactive endothelin in rat kidney inner medulla: marked decrease in spontaneously hypertensive rats. Biochem Biophys Res Commun.

[22] Wu F, Park F, Cowley AW, Mattson DL (1999). Quantification of nitric oxide synthase activity in microdissected segments of the rat kidney. Am J Physiol.

[23] Hyndman KA (2013). Renal collecting duct NOS1 maintains fluid-electrolyte homeostasis and blood pressure. Hypertension.

[24] Hyndman KA (2017). Collecting duct nitric oxide synthase 1ß activation maintains sodium homeostasis during high sodium intake through suppression of aldosterone and renal angiotensin II pathways. J Am Heart Assoc.

[25] Lee JW, Chou CL, Knepper MA (2015). Deep sequencing in microdissected renal tubules identifies nephron segment-specific transcriptomes. J Am Soc Nephrol.

[26] Chen S, Yao X, Li Y, Saifudeen Z, Bachvarov D, El-Dahr SS (2015). Histone deacetylase 1 and 2 regulate Wnt and p53 pathways in the ureteric bud epithelium. Development.

[27] Hyndman KA, Kasztan M, Mendoza LD, Monteiro-Pai S (2019). Dynamic changes in histone deacetylases following kidney ischemia-reperfusion injury are critical for promoting proximal tubule proliferation. Am J Physiol Renal Physiol.

[28] Bolden JE, Peart MJ, Johnstone RW (2006). Anticancer activities of histone deacetylase inhibitors. Nat Rev Drug Discov.

[29] Hoffert JD, Pisitkun T, Wang G, Shen RF, Knepper MA (2006). Quantitative phosphoproteomics of vasopressin-sensitive renal cells regulation of aquaporin-2 phosphorylation at 2 sites. Proc Natl Acad Sci U S A.

[30] Ahn D (2004). Collecting duct-specific knockout of endothelin-1 causes hypertension and sodium retention. J Clin Invest.

[31] Gao M (2015). Disruption of prostaglandin E2 receptor EP4 impairs urinary concentration via decreasing aquaporin 2 in renal collecting ducts. Proc Natl Acad Sci U S A.

[32] Baumann M (2011). Urinary nitric oxide metabolites and individual blood pressure progression to overt hypertension. Eur J Cardiovasc Prev Rehabil.

[33] Schönig K, Schwenk F, Rajewsky K, Bujard H (2002). Stringent doxycycline dependent control of CRE recombinase in vivo. Nucleic Acids Res.

[34] Traykova-Brauch M (2008). An efficient and versatile system for acute and chronic modulation of renal tubular function in transgenic mice. Nat Med.

[35] Jespersen H (2019). Concomitant use of pembrolizumab and entinostat in adult patients with metastatic uveal melanoma (PEMDAC study): protocol for a multicenter phase II open label study. BMC Cancer.

[36] Jiang Z (2019). Tucidinostat plus exemestane for postmenopausal patients with advanced, hormone receptor-positive breast cancer (ACE): a randomised, double-blind, placebo-controlled, phase 3 trial. Lancet Oncol.

[37] Krug LM (2015). Vorinostat in patients with advanced malignant pleural mesothelioma who have progressed on previous chemotherapy (VANTAGE-014): a phase 3, double-blind, randomised, placebo-controlled trial. Lancet Oncol.

[38] Joergensen D, Tazmini K, Jacobsen D (2019). Acute Dysnatremias - a dangerous and overlooked clinical problem. Scand J Trauma Resusc Emerg Med.

[39] Tazmini K, Nymo SH, Louch WE, Ranhoff AH, Øie E (2019). Electrolyte imbalances in an unselected population in an emergency department: A retrospective cohort study. PLoS One.

[40] Paice BJ, Paterson KR, Onyanga-Omara F, Donnelly T, Gray JM, Lawson DH (1986). Record linkage study of hypokalaemia in hospitalized patients. Postgrad Med J.

[41] Han WK, Bailly V, Abichandani R, Thadhani R, Bonventre JV (2002). Kidney Injury Molecule-1 (KIM-1): a novel biomarker for human renal proximal tubule injury. Kidney Int.

[42] Avissar N (1994). Human kidney proximal tubules are the main source of plasma glutathione peroxidase. Am J Physiol.

[43] Roxborough HE, Mercer C, McMaster D, Maxwell AP, Young IS (1999). Plasma glutathione peroxidase activity is reduced in haemodialysis patients. Nephron.

[44] Pang P (2018). Pre-clinical model of severe glutathione peroxidase-3 deficiency and chronic kidney disease results in coronary artery thrombosis and depressed left ventricular function. Nephrol Dial Transplant.

[45] Liu H (2018). Histone deacetylases 1 and 2 regulate the transcriptional programs of nephron progenitors and renal vesicles. Development.

[46] Ledo N (2015). Functional genomic annotation of genetic risk loci highlights inflammation and epithelial biology networks in CKD. J Am Soc Nephrol.

[47] Maroz N, Maroz U, Iqbal S, Aiyer R, Kambhampati G, Ejaz AA (2012). Nonobstructive hydronephrosis due to social polydipsia: a case report. J Med Case Rep.

[48] Streitz JM, Streitz JM (1988). Polyuric urinary tract dilatation with renal damage. J Urol.

[49] Tsai SC, Valkov N, Yang WM, Gump J, Sullivan D, Seto E (2000). Histone deacetylase interacts directly with DNA topoisomerase II. Nat Genet.

[50] Johnson CA, Padget K, Austin CA, Turner BM (2001). Deacetylase activity associates with topoisomerase II and is necessary for etoposide-induced apoptosis. J Biol Chem.

[51] Skok Ž, Zidar N, Kikelj D, Ilaš J (2020). Dual inhibitors of human DNA topoisomerase II and other cancer-related targets. J Med Chem.

[52] Li S (2018). Ectodysplasin A regulates epithelial barrier function through sonic hedgehog signalling pathway. J Cell Mol Med.

[53] Zhang R (2020). End-stage renal disease is different from chronic kidney disease in upregulating ROS-modulated proinflammatory secretome in PBMCs - A novel multiple-hit model for disease progression. Redox Biol.

[54] Lee HA (2018). Histone deacetylase inhibition ameliorates hypertension and hyperglycemia in a model of Cushing’s syndrome. Am J Physiol Endocrinol Metab.

[55] Cavasin MA, Stenmark KR, McKinsey TA. Emerging roles for histone deacetylases in pulmonary hypertension and right ventricular remodeling (2013 Grover Conference series). *Pulm Circ*. 2015;5(1):63-72 10.1086/679700 PMC4405717 25992271

[56] Choi J, Park S, Kwon TK, Sohn SI, Park KM, Kim JI (2017). Role of the histone deacetylase inhibitor valproic acid in high-fat diet-induced hypertension via inhibition of HDAC1/angiotensin II axis. Int J Obes (Lond).

[57] Armas-Padilla MC (2007). Nitric oxide and malondialdehyde in human hypertension. Am J Ther.

[58] Bachmann S, Bosse HM, Mundel P (1995). Topography of nitric oxide synthesis by localizing constitutive NO synthases in mammalian kidney. Am J Physiol.

[59] Lyamina NP, Dolotovskaya PV, Lyamina SV, Malyshev IY, Manukhina EB (2003). Nitric oxide production and intensity of free radical processes in young men with high normal and hypertensive blood pressure. Med Sci Monit.

[60] Mattson DL, Bellehumeur TG (1996). Neural nitric oxide synthase in the renal medulla and blood pressure regulation. Hypertension.

[61] Imanishi M (2013). Angiotensin II receptor blockade reduces salt sensitivity of blood pressure through restoration of renal nitric oxide synthesis in patients with diabetic nephropathy. J Renin Angiotensin Aldosterone Syst.

[62] Hsu CN, Lu PC, Lo MH, Lin IC, Tain YL (2019). The association between nitric oxide pathway, blood pressure abnormalities, and cardiovascular risk profile in pediatric chronic kidney disease. Int J Mol Sci.

[63] Hsu CN, Tain YL (2019). Regulation of nitric oxide production in the developmental programming of hypertension and kidney disease. Int J Mol Sci.

[64] Kemmner S (2017). Dietary nitrate load lowers blood pressure and renal resistive index in patients with chronic kidney disease: A pilot study. Nitric Oxide.

[65] D’Angelo G, Pollock JS, Pollock DM (2006). In vivo evidence for endothelin-1-mediated attenuation of alpha1-adrenergic stimulation. Am J Physiol Heart Circ Physiol.

[66] Speed JS, Hyndman KA (2016). In vivo organ specific drug delivery with implantable peristaltic pumps. Sci Rep.

[67] Pawlowska D, Granger JP, Knox FG (1987). Effects of adenosine infusion into renal interstitium on renal hemodynamics. Am J Physiol.

[68] Saito A (1999). A synthetic inhibitor of histone deacetylase, MS-27-275, with marked in vivo antitumor activity against human tumors. Proc Natl Acad Sci U S A.

[69] Yang X, Zhang Q, Chen M, Hu L (2014). Pharmacokinetic interaction of entinostat and lapatinib following single and co-oral administration in rats. Xenobiotica.

[70] Wu H, Kirita Y, Donnelly EL, Humphreys BD (2019). Advantages of single-nucleus over single-cell RNA sequencing of adult kidney: rare cell types and novel cell states revealed in fibrosis. J Am Soc Nephrol.

[71] Efthimiou O (2018). Practical guide to the meta-analysis of rare events. Evid Based Ment Health.

