# Supplementary material for: Fluid-electrolyte homeostasis requires histone deacetylase function
Source: JCI Insight. 2020 Aug 20;5(16):e137792. doi: 10.1172/jci.insight.137792 (PMC7455138; doi:10.1172/jci.insight.137792)
Supplement: Supplemental data [file jciinsight-5-137792-s033.pdf]

Supporting Information

Hyndman et al.

Rats, telemetry and chronic intramedullary infusion. All animal use and welfare adhered to the NIH Guide for the Care and Use of Laboratory Animals following a protocol reviewed and approved by the Institutional Laboratory Animal Care and Use Committee of The University of Alabama at Birmingham. Sprague Dawley, 8-week-old male rats (225 g) were purchased from Harlan (Indianapolis, IN) and maintained on a 12 h light 12 h dark schedule. These rats were fed a normal salt diet (0.49% NaCl Teklad #96208) and water ad libitum. At 10 weeks of age, rats (N = 5) were randomly assigned to either normal salt diet or high salt (4.0% NaCl Teklad #92034) for 7 days. These rats were then euthanized and plasma, kidney cortex, outer medulla and inner medulla were dissected and snap frozen for Western blot experiments.

In two additional groups of rats (n= 5 per vehicle or MS275 for each protocol, for a total of 20 rats undergoing this surgery), at 9 weeks of age underwent uninephrectomy, leaving the right kidney intact, and implanted with telemetry devices (Data Sciences Inc, St. Paul, MN) as previously described (1). These rats were allowed to recover for 1 week then the transmitters were turned on for blood pressure, heart rate and activity measurements prior to the implantable peristaltic pump surgery listed below.

Prior to surgery, sterile iPRECIO® micro infusion pumps (SMP-200, Tokyo, Japan) were filled with sterile warmed 0.9% saline, and placed at 37°C for 4 h. After this, the pumps were programmed to deliver saline at a rate of 30 μl/min for 30 min in

order to keep the catheter patent during surgery and then set to infuse at a rate of 9 µl/min for the duration of the study. This rate of delivery was based upon previous studies that determined 10 µl/min infusion of 0.9% saline had no significant effect on renal hemodynamics (2). These pumps were refilled percutaneously with fresh solutions (see below) on every 4th day.

Next, the rats were anesthetized with 2% isoflurane, and given a s.c. injection of buprenorphine (0.1 mg/kg) and carprofen (5 mg/kg) to minimize any potential surgical pain. A flank incision was made over the right kidney and it was implanted with a chronic indwelling catheter of v-1 tubing connected to the tubing of the peristaltic pump with superglue. The iPRECIO® micro infusion pump was placed in a subcutaneous pocket on the dorsal surface of the rat, and the catheter tunneled through the muscle into the abdomen. The catheter was then placed in the middle of a 5 mm round piece of alliedsil (Allied Biomedical, Paso Robles, CA) sheeting, and the catheter inserted 4-5 mm into the kidney secured to the renal capsule with vetbond. The muscle was then sutured together, and the skin stapled together. 0.25% marcaine + 0.5% lidocaine (mixed 50/50) was given locally on the incision. The rats were allowed to recover in clean cages for 48 h before being placed individually into metabolic cages. During the course of the study, 1 vehicle rat died and his samples were excluded from all analyses.

Urine, Plasma and Tissue Collection. During Infusion protocol 1 and 2, urine was collected every 24 h. At the end of infusion protocol 1 and 2, plasma samples were taken, and the kidneys dissected into cortex, outer and inner medulla. Aliquots of urine, plasma and the kidney parts were snap frozen and stored at -80°C.

Urine and Plasma Analyses. Urine samples from experimental days 2 and 7 were analyzed for sodium and potassium by atomic absorption (Perkin Elmer, Waltham, MA), osmolality (Vapor Pressure Osmometer, Elitech Group Solutions, Princeton, NJ), urea (BioAssay Systems, Hayward, CA), and protein (Quickstart ®, Biorad, Carlsbad, CA). Creatinine was measured by mass spectrometry by the UAB-UCSD O'Brien Center Bioanalytical Core (Birmingham, AL).

Plasma sodium, potassium and chloride were measure by ion-selective electrodes (Easylyte, Medica, Bedford, MA), osmolality with a vapor pressure osmometer, and urea by assay. Creatinine was measured by mass spectrometry by the UAB-UCSD O'Brien Center Bioanalytical Core (Birmingham, AL).

Urinary and plasma aldosterone was measure by EIA (Cayman Chemical), urinary vasopressin by ELISA (Enzo), urinary ANP by EIA (Peninsula Laboratories, Can Carlos, CA), urinary ET-1 by ELISA (QuantiGlo®, R&D Systems), urinary nitrite/nitrate (NOx) by HPLC (ENO-30, Eicom, CA). Plasma renin concentration was measured as previously described (3).

Murine Models

Whole nephron Hdac1 and Hdac2 Knockout. *Hdac1*$^{fl/fl}$ and *Hdac2*$^{fl/fl}$ mice (CD-1 strain) (4) were bred with *Pax8-rtTA* (Jax Labs stock #007176, C57BL/6J, (5) */Lc-1-Cre* (6) mice until all mice were homozygous for *Hdac1*$^{fl/fl}$ and *Hdac2*$^{fl/fl}$. Initially, all mice were provided 2 mg/ml doxycycline in 2% sucrose water for 14 days, followed by 14-day washout. Mice positive for *Pax8-rtTA/LC-1* died by day 27 (males, n = 6) and day 28

(females, n = 6) post initiation of doxycycline. Control mice, with either only homozygous floxed or had either *Pax8-rtTA, or LC-1* (but not both) all survived. Next, we used only 1 week of doxycycline water with 1 week of washout. All mice survived. Blood and kidney samples were taken at this time point (see details below).

Collecting duct Hdac1 and Hdac2 Knockout (iHoxb7 Hdac1/2KO). Hdac1$^{fl/fl}$ and *Hdac2$^{fl/fl}$* mice (CD-1) were bred with *Hoxb7-rtTA* (Jax Labs Stock #016567, mixed background (7)*/Lc-1-Cre* mice until all mice were homozygous for *Hdac1$^{fl/fl}$* and *Hdac2$^{fl/fl}$*. Mice were provided 2 mg/ml doxycycline in 2% sucrose water and doxycycline diet (Envigo, TD.08434) for 14 days, followed by 14-day washout.

Collecting duct Hdac1 knockout. Hdac1$^{fl/fl}$ and *Hdac2$^{fl/fl}$* mice were bred with C57BL/6J mice, and mice only *Hdac1$^{fl/wt}$ Hdac2$^{wt/wt}$* inbred to generate *Hdac1$^{fl/fl}$* homozygotes. *Hdac1$^{fl/fl}$* were then bred with *Hoxb7-rtTA/Lc-1-Cre* until all mice were homozygous for *Hdac1$^{fl/fl}$.* Mice were provided 2 mg/ml doxycycline in 2% sucrose water and doxycycline diet (Envigo, TD.08434) for 14 days, followed by 14-day washout.

Mouse metabolic cages and telemetry. Because the *iPax8 Hdac1/2KO* mice were not healthy, they were only studied on an ad lib normal salt diet at 2 weeks post knockdown. The collecting duct specific *Hoxb7* colonies, both controls and knockouts of both sexes were individually housed in metabolic cages as previously described (8, 9). Mice were provided gel diet and acclimated to the cages for 48 h before sampling occurred (9). Low salt diet had 0.01% NaCl (LS), normal salt had 0.4% NaCl, and high

salt had 4.0% NaCl (HS). All other dietary components were the same. Mice were provided water ad libitum. Urine (collecting under water saturated mineral oil), food and water intake, were collected every 24 h at 9 am. Urine samples were centrifuged 1000 g for 10 min to pellet any sediment, and the liquid was aliquoted and stored at -80°C until analysis. Urine was analyzed for electrolytes and osmolality as outlined in the rat protocol above. Mice were kept on a LS diet for a week, followed by 6, 24-hour periods of HS, and 3 24 hour periods of NS. After the metabolic cage protocol, mice remained individually housed in regular cages for 1 week. Then they were implanted with telemetry devices as previously described (10). Mice again went through the dietary salt intervention by providing the gel diet daily. Blood pressure, heart rate, and activity were recorded. After this, mice were randomly assigned to either LS or HS treatment groups for 1 week and samples taken as described below.

Mouse blood, and tissue collections. Mice were anesthetized with inhaled 2% isoflurane and once unresponsive, blood was taken by cardiac puncture through the diaphragm. Immediately, blood was analyzed with the iSTAT EC8+ cartridge (Abbott Labs). The rest of the blood was centrifuged at 1000 g for 10 min and plasma snap frozen and stored at -80°C. The kidneys were excised, decapsulated, and the left kidney cut in cross section for histological analysis. Kidneys were instantly placed in 10% neutral buffered formalin for 24 h at room temperature, and then stored in 70% ethanol until embedding in paraffin wax. The right kidney was divided into cortex, outer medulla and inner medulla samples and snap frozen for molecular analysis listed below. In the *iHoxb7 Hdac1/2KO* and control mice study, 11/30 KO mice had a gross kidney

abnormality (hydronephrosis, atrophied, or only a single kidney). In this case samples were only taken for histological analysis.

Confirmation of mouse genotypes and recombination of floxed alleles:
Genotyping was performed with DNA extracted from tails snips. Primers are listed in **Table S11.** Starting in 2018 mouse genotyping was performed by Transnetyx (Cordova, TN).

PCR for recombinant alleles from DNA isolated from kidney samples (Sigma GenElute G1N70) was run with primers listed in **Table S11**. DNA was nanodropped (NanoDrop One^{c}, Thermo Fisher Scientific) and diluted to 30 μg/μl in RNAse-DNAse free water. The master mix for PCR was made using 12.5 μl 2x DreamTaq Hot Start Green PCR Mastermix (K9022, Thermo Fisher Scientific), 9μl of RNAse-DNAse free water, and 0.5 μl each of 10 μM Forward primer, 10μM Reverse primer, and 10μM Recombinant primer for either HDAC1 or HDAC2 and 2 μl of 30 μg/μl DNA per sample. All samples were run in a thermocycler (T100 Thermal Cycler, BIO-RAD) with the following recombinant allele protocols: For HDAC1 (1) 95°C, 5 minutes, (2) 95°C, 30 seconds, (3) 57°C, 30 seconds, (4) 72°C, 45 seconds, (5) Repeat step 2-4 37 times, (6) 72°C, 7 minutes. HDAC2 recombinant thermocycler protocol: (1) 95°C, 5 minutes, (2) 95°C, 30 seconds, (3) 61°C, 30 seconds, (4) 72°C, 45 seconds, (5) Repeat step 2-4 37 times, (6) 72°C, 7 minutes. PCR products were visualized with 1.5% agarose GPG/LE (AB00972-00500, AmericanBIO) gels made with 0.5X Tris-acetic acid-EDTA buffer (#B49, Thermo Fisher Scientific) and GreenGlo (CA3600, Denville Scientific Inc.), and

run in a wide Mini-Sub Cell GT (Bio-Rad) for 35 minutes at 100 volts. Gels were imaged using the Thermo Fisher Scientific E-Gel Imager under blue light.

Mouse kidney histology and immunohistochemistry. The kidneys were embedded in paraffin wax and cut into 5 micron sections and placed on superfrost plus slides (Fisher). Slides were processed as described in detail (8) using Gomori's trichrome staining (Richard Allan). All samples were blinded to investigators. Each kidney was examined and any protein casts, interstitial fibrosis, dilated tubules, or atrophied tubules recorded.

To determine if HDAC1 and/or HDAC2 were knocked out in the kidney epithelial, immunohistochemistry was performed. Antibodies are listed in **Table S12**. Slide processing was reported in (8). HDAC1 and HDAC2 primary antibodies were left on the sections overnight at 4°C, and visualized with horse radish-peroxidase coupled secondary antibodies and 3,3-Diaminobenzidine (Vector Labs). Slides were counterstained for 1 min with hematoxylin (Gill 2 hematoxylin, Ricca, 3536-32)

Western Blots. Human kidney lysate samples were purchased from samples Origene (Rockville, MD) under strict IRB and ethical consenting practices. Sample characteristics were previously described (11). mIMCD-3, cortex, outer medulla and inner medulla samples were homogenized in 10 vols/ wt of lysis buffer (20 mM Tris, 150 mM NaCl, 0.5% Triton X-100, 1 mM EDTA, 1 mM EGTA, 10 μ□M leupeptin, 1 mM phenylmethylsulfonyl fluoride, pH 7.5) + 1 Phosphatase inhibitor mini tab/10 ml lysis (Pierce Thermo Scientific). For total cell lysates, the sample was spun at 1000 g for 1

min to pellet cell debris. For histone extraction, the lysates were spun at 6,500 x g for 10 min to pellet nuclei. Histones were then extracted from the nuclear pellet by acid extraction with 5 volumes of 0.2 N hydrochloric acid, overnight at 4°C. The histone sample was then spun at 6,500 g for 10 min at 4°C to pellet debris, and the protein concentration of the histone supernatant and original lysate determined by Bradford assay (Quickstart®, Biorad, Carlsbad, CA), and samples stored at -20°C until used in Westerns. Proteins were separated with 8% or 15% SDS PAGE and transferred to PVDF membranes. Antibodies used in the study are listed in **Table S12**, and were placed on the membrane overnight at 4°C. Primary antibodies were visualized with fluorescent tagged secondary antibodies (AlexaFluor® 680 or 800, Thermo Fisher) and imaged using the Odyssey Infrared Imaging System (Licor Biosciences, Lincoln, NE). All normalizations (for example to actin, total NOS3, total H3) were performed on the same membrane and visualized using different fluorescent tags.

Single Nuclear RNA isolation and single nucleus sequencing (snRNA-Seq). *iPax8 Hdac1/2KO* and control both male and female mice, were anesthetized with inhaled 2% isoflurane, a cardiac puncture blood sample taken and immediately analyzed for electrolytes using an iSTAT EC8+ cartridge. Ten milliliters of sterile 10 mM phosphate buffered saline was delivered through the heart to perfuse the kidneys. The kidneys were then immediately excised, decapsulated, and the left kidney cut into 5 mm cubes and snap frozen. Half of the right kidney was snap frozen, and the other half fixed for histological analyses as stated above.

Single nuclei suspensions of the whole kidney were isolated as previously described (12). In addition to the protease inhibitor, cOmplete™ ULTRA tab (Sigma), 2 RNase inhibitors were used in the Nuclei EZ Lysis buffer (Sigma): 1) RNAsin Plus (5 μl/ml, Promega, Madison, WI) and 2) SUPERaseIN (5 μl/ml , ThermoFisher). The frozen kidney cubes were placed in a sterile petri dish with 1 ml of Nuclei EZ lysis buffer (plus protease and RNase inhibitors) and quickly minced. The tissue was then homogenized with RNase-free, disposable pellet pestles and tubes (Fisher #12-141-368), and a hand held, Bel-Art™ Micro-Tube homogenizer (Wayne, NJ). The homogenate was filtered through a 200 micron strainer (all strainers were purchase from pluriSelect Life Science), and homogenized again with a fresh tube and pestle. The homogenate was moved to a sterile, RNase-free 15 ml tube with an additional 2 ml of Nuclei EZ lysis buffer (plus protease and RNase inhibitors). After a 5 min incubation on ice, the homogenate was filtered through a 40 micron strainer. The subsequent homogenate was centrifuged at 500 g for 5 min at 4°C. The pellet was resuspending in Nuclei EZ lysis buffer (plus 1 μl/ml of SUPERaseIN and RNAsin), incubated on ice for 5 min and centrifuged again. The pellet was resuspended in 2 ml of 1X Dulbecco's Phosphate-buffered saline + 1% bovine serum albumin, filtered through a 5 micron strainer, and nuclei immediately counted with a hemocytometer. At least 5000 nuclei from each sample were placed into oil droplets with the 10X Genomics Chormium™ Single Cell B Chip kit, and cDNA libraries made using 10x Chorminum™ Single cell 3' library and gel bead kit (version 3, 10X Genomics). Single nuclear cDNA libraries were sequenced in collaboration with the UAB Genomics Core with an Illumina Nextseq500 machine with 20,000 reads/cell at minimum. Fastq files were aligned and counted using

CellRanger v3.0.2 using the pre-mRNA mouse mm10 reference genome (10X Genomics). Raw and processed files are deposited in GEO Accession number pending.

Unbiased clustering and cell type annotation from snRNA-Seq dataset. The count matrices were further analyzed with R version 3.6.2 and the package *Seurat* version 3.0 (13). Seurat objects for each of the four samples were created with a minimum of 3 cells and RNA features of > 500 per nuclei to filter low quality nuclei. Next, the nuclei were filtered to exclude samples with < than 2% of mitochondrial genes. The Seurat objects were then normalized using *SCTransform* and combined into a single list with groupid = “KO” or “Con” and sex = “male” or “female”. To start to integrate the dataset, we used *SelectIntegrationFeatures* nfeatures = 3000, and *PrepSCTIntegration*. Anchors were found using *FindIntegrationAnchors*, normalization.method = “SCT”, and integrated using the command *IntegrateData*. Dimensionality reduction was performed with Principal Components Analysis (PCA) followed by Uniform Manifold Approximation and Projection (UMAP) with dims = 1:20. *FindNeighbors* and *FindClusters* with a resolution = 0.2 was used to identify clusters. Specific cluster markers (positive and negative expression) were identified using *FindAllMarkers*, with a minimum percent nuclei expression = 0.25, and a log fold change (logFC) >0.25. The workflow identified 18 clusters, and using published markers the cell types were identified by manual review.

Differentially expressed Genes (DEGs). DEGs within each cluster comparing "Con" to "KO" (sexes combined) were identified using the *LogNormalized* data found in the "SCT" assay of the Seurat objects and the command *FindMarkers*. Volcano plots of DEGs were created using *EnhancedVolcano*. Heatmaps of genes of interest were generated using average logFC and adjusted P-values (for multiple comparisons) in Prism (version 8.3.1). Cluster 12 (named PT5) was unique to the KO animals. Downstream analyses of Cluster 12 compared to all other clusters, proximal tubule clusters (PT1-4), or immune cell clusters (Macrophages and Immune) were performed using *Findmarkers* between these clusters. The significant markers enriched in Cluster 12 were analyzed using DAVID Bioinformatics Resource 6.8 (National Institutes of Health). Heatmaps were generated with Prism (Graphpad, v8.3.1), and significant gene interactions in Gene Ontology Biological Processes were plotted with *GoChord* in the R package GoPlot (14).

Systematic Review and meta-analysis. In August 2019, literature searches were performed on Pubmed and Clinicaltrials.gov. Search terms are outlined in **Figure S15**. Inclusion criteria included any histone deacetylase inhibitor (HDACi) compared to either a placebo or standard of care. Adverse events separated by grades 1-5 and including plasma or blood electrolyte measurements were included for further analysis. As outlined in **Figure S15**, there were 12 studies that met our inclusion criteria. From this dataset we analyzed only serious adverse events, as defined as ≥ grade 3, Fluid-electrolyte disorders and changes in pressure. Fluid-electrolyte disorder was defined as any ≥ grade 3 event reported for the following either hypo- or hyper-: glycaemia,

albuminemia, calcaemia, kalemia, natremia, phosphatemia, uricaemia, magnesemia. For each study, each event was summed to give the total number of fluid-electrolyte disorder events for the HDACi and placebo/standard of care comparison. Three other datasets were also analyzed for ≥ grade 3 events: 1) hyponatremia, 2) hypokalemia, 3) Change in blood pressure (summation of events recorded as hypo- or hyper-tension). These datasets were analyzed with R (15) using R Studio (16) and the Mantel-Haenszel method following the code of Efthimiou (17). This method considers rare and zero count events. Both fixed and random effects models are reported. Results from the analysis were represented by forest plots of odds ratios and 95% confidence intervals, generated in R. In **figures S16, S17**, event counts, weights, tests of heterogeneity, are reported.

Literature Cited in Supporting Information

1. D'Angelo G, Pollock JS, and Pollock DM. In vivo evidence for endothelin-1-mediated attenuation of alpha1-adrenergic stimulation. *American journal of physiology Heart and circulatory physiology.* 2006;290(3):H1251-8.
2. Pawlowska D, Granger JP, and Knox FG. Effects of adenosine infusion into renal interstitium on renal hemodynamics. *The American journal of physiology.* 1987;252(4 Pt 2):F678-82.
3. Ramkumar N, Stuart D, Rees S, Van Hoek AN, Sigmund CD, and Kohan DE. Collecting duct specific knock-out of renin attenuates angiotensin-II induced hypertension. *American journal of physiology Renal physiology.* 2014.
4. Montgomery RL, Davis CA, Potthoff MJ, Haberland M, Fielitz J, Qi X, et al. Histone deacetylases 1 and 2 redundantly regulate cardiac morphogenesis, growth, and contractility. *Genes Dev.* 2007;21(14):1790-802.
5. Traykova-Brauch M, Schonig K, Greiner O, Miloud T, Jauch A, Bode M, et al. An efficient and versatile system for acute and chronic modulation of renal tubular function in transgenic mice. *Nat Med.* 2008;14(9):979-84.
6. Schonig K, Schwenk F, Rajewsky K, and Bujard H. Stringent doxycycline dependent control of CRE recombinase in vivo. *Nucleic acids research.* 2002;30(23):e134.
7. Shakya R, Jho EH, Kotka P, Wu Z, Kholodilov N, Burke R, et al. The role of GDNF in patterning the excretory system. *Developmental biology.* 2005;283(1):70-84.
8. Hyndman KA, Kasztan M, Mendoza LD, and Monteiro-Pai S. Dynamic changes in histone deacetylases following kidney ischemia-reperfusion injury are critical for promoting proximal tubule proliferation. *American journal of physiology Renal physiology.* 2019;316(5):F875-F88.
9. Hyndman KA, Mironova EV, Giani JF, Dugas C, Collins J, McDonough AA, et al. Collecting Duct Nitric Oxide Synthase 1 Activation Maintains Sodium Homeostasis During High Sodium Intake Through Suppression of Aldosterone and Renal Angiotensin II Pathways. *J Am Heart Assoc.* 2017;6(10).
10. Hyndman KA, Boesen EI, Elmarakby AA, Brands MW, Huang P, Kohan DE, et al. Renal Collecting Duct NOS1 Maintains Fluid-Electrolyte Homeostasis and Blood Pressure. *Hypertension.* 2013;62:91-8.
11. Hyndman KA, Arguello AM, Morsing SK, and Pollock JS. Dynamin-2 is a novel NOS1beta interacting protein and negative regulator in the collecting duct. *Am J Physiol Regul Integr Comp Physiol.* 2016;310(7):R570-7.
12. Wu H, Kirita Y, Donnelly EL, and Humphreys BD. Advantages of Single-Nucleus over Single-Cell RNA Sequencing of Adult Kidney: Rare Cell Types and Novel Cell States Revealed in Fibrosis. *Journal of the American Society of Nephrology : JASN.* 2019;30(1):23-32.
13. Stuart T, Butler A, Hoffman P, Hafemeister C, Papalexi E, Mauck WM, 3rd, et al. Comprehensive Integration of Single-Cell Data. *Cell.* 2019;177(7):1888-902 e21.
14. Walter W, Sanchez-Cabo F, and Ricote M. GOplot: an R package for visually combining expression data with functional analysis. *Bioinformatics.* 2015;31(17):2912-4.
15. Team RC. 2017.

16. Team RS. 2015.
17. Efthimiou O. Practical guide to the meta-analysis of rare events. *Evid Based Ment Health.* 2018;21(2):72-6.

Table S1: Twenty four hour blood pressure and heart rate data from the rat and mouse studies. Results of Student's *t*-test reported in *P* value (bold indicates P<0.05).

		Systolic			Diastolic			MAP			Heart Rate			Activity		
**Rats all male**	***n***	**mean**	**s.e.m.**	**P value**	**mean**	**s.e.m.**	**P value**	**mean**	**s.e.m.**	**P value**	**mean**	**s.e.m.**	**P value**	**mean**	**s.e.m.**	**P value**
Vehicle HSD7	5	141.80	5.10	**0.04**	92.43	4.68	**0.02**	115.00	4.71	**0.02**	390.50	3.81	**0.01**	n.d.		
MS275HSD7	5	154.40	2.12		106.70	1.78		128.50	2.00		355.20	8.54		n.d.		
Vehicle HSW7	4	129.90	1.92	**0.01**	89.92	2.03	**0.01**	109.50	2.01	**0.02**	388.50	2.94	**0.03**	2.00	0.29	0.84
MS275HSW7	5	141.40	3.09		99.10	1.87		118.60	2.26		373.90	4.89		2.08	0.27	
**Mice all HS6**																
Control Male	10	138.30	2.99	0.96	96.81	2.41	0.17	117.80	2.35	0.45	527.90	7.67	0.67	4.93	0.65	0.94
iHoxb7 Hdac1/2KO male	10	138.60	5.03		102.50	3.11		121.20	3.70		523.20	7.84		4.99	0.52	
Control Female	11	131.90	1.82	0.62	98.52	1.89	0.99	116.20	1.71	0.76	584.70	6.93	0.08	9.36	0.93	**0.02**
iHoxb7 Hdac1/2KO Female	7	130.50	2.24		98.48	3.66		115.20	2.16		560.90	11.60		5.33	1.17	
Control both sexes	11	128.00	4.34	0.47	93.34	2.48	0.91	111.40	3.33	0.57	562.30	5.46	0.44	3.69	0.62	0.68
i iHoxb7 Hdac1 KO both sexes	9	123.70	3.75		92.95	2.42		108.70	3.05		571.30	10.84		3.34	0.53	

Table S2: Metabolic cage and excretion data from rats on a high salt diet (HSD) for 2 or 7 days with either an intramedullary vehicle infusion or class I HDAC inhibitor, MS275. * represents $P < 0.05$ from post hoc Sidak's multiple comparison between vehicle and MS275.

	Vehicle				MS275				Two Factor ANOVA		
	HSD2		HSD7		HSD2		HSD7		P_{diet}	P_{drug}	P_{DxD}
Sample size	4		4		5		5				
	mean	s.e.m.	mean	s.e.m.	mean	s.e.m.	mean	s.e.m.			
Food intake, g/day	17.60	0.61	16.80	1.14	14.70	1.99	19.30	1.69	0.22	0.90	0.10
Sodium intake, g/day	0.27	0.02	0.34	0.03	0.18	0.05	0.30	0.01	**0.02**	0.08	0.76
Water intake, ml/day	54.43	4.38	51.38	2.93	49.74	2.98	**68.40***	**6.42**	0.07	0.30	**0.02**
Urine Flow , ml/day	32.29	2.85	34.41	2.00	28.42	3.40	**49.80***	**5.04**	**0.01**	0.22	**0.02**
UNaV, mmol/day	11.00	0.88	9.20	0.73	8.30	2.00	7.40	0.91	0.35	0.16	0.73
UKV, mmol/day	3.03	0.33	2.60	0.23	2.70	0.44	1.90	0.26	0.07	0.22	0.52
Urea, mg/ml	27.83	1.58	27.10	3.10	29.80	2.56	**17.10***	**2.93**	**0.02**	0.24	**0.03**
UureaV, mg/day	885.48	35.16	916.86	64.54	836.12	114.77	824.38	114.02	0.86	0.59	0.71
Aldosterone, ng/day	752.02	135.86	1038.80	114.98	1092.00	126.93	1196.60	172.56	**0.03**	0.23	0.24
ANP, ng/day	43.16	13.17	46.22	18.98	39.44	17.10	33.37	12.46	0.92	0.60	0.78
Vasopressin, ng/day	2.37	0.93	2.03	0.65	1.63	0.30	**5.01***	**0.28**	**0.03**	0.09	**0.01**
PGEM, ng/day	11.87	0.29	15.88	2.61	8.49	1.56	18.30	2.82	**0.01**	0.83	0.20
ET-1, pg/day	7.23	1.51	7.98	1.36	7.60	1.80	**15.93***	**1.75**	**0.02**	0.05	**0.05**
H_2O_2, µmol/day	0.95	0.15	1.70	0.29	0.91	0.28	1.30	0.42	0.14	0.61	0.55
										T-test P value	
CH_2O, ml/h	n.d.	n.d.	-4.20	0.20	n.d.	n.d.	-2.70	0.41		**0.019**	
Creatinine Clearance, ml/min/100 g B.W.	n.d.	n.d.	0.62	0.06	n.d.	n.d.	0.68	0.05		0.67	

Table S3: Metabolic cage data, excretion and plasma measurements from rats on 1% high NaCl water (HSW) for 2 or 7 days with either an intramedullary vehicle infusion or class I HDAC inhibitor, MS275. . * represents P < 0.05 from post hoc Sidak's multiple comparison between vehicle and MS275.

	Vehicle				MS275				Two Factor ANOVA		
	HSW2		HSW7		HSW2		HSW7		P_{diet}	P_{drug}	P_{DxD}
Sample size	5		5		5		5				
	mean	s.e.m.	mean	s.e.m.	mean	s.e.m.	mean	s.e.m.			
Food intake, g/day	18.82	0.64	20.57	0.67	20.52	0.76	18.58	0.36	0.83	0.85	**0.002**
Sodium intake, g/day	0.25	0.01	0.27	0.02	0.29	0.03	0.31	0.03	0.16	0.27	0.69
Water intake, ml/day	53.52	1.47	57.65	5.30	62.41	6.55	68.37	6.41	0.17	0.14	0.78
Urine Flow , ml/day	37.85	2.60	41.44	2.39	42.70	5.10	51.42	5.50	**0.009**	0.22	0.19
UNaV, mmol/day	9.10	0.46	10.21	0.54	11.65	0.74	**17.21***	**1.64**	**0.005**	**0.002**	**0.038**
UKV, mmol/day	2.40	0.14	2.74	0.31	2.27	0.15	2.94	0.21	**0.030**	0.86	0.44
Urea, mg/ml	38.44	6.17	58.70	7.41	33.70	7.63	33.76	4.35	0.12	0.07	0.17
UureaV, mg/day	1418.47	192.81	2366.48	178.01	1331.29	290.17	1648.66	114.10	**0.013**	0.088	0.15
Uosmolality, mOsm/kg H_2O	1249.20	106.28	915.80	60.74	**890.80***	**89.80**	819.00	73.50	**0.007**	0.063	**0.049**
Aldosterone, ng/day	970.35	137.90	960.59	120.76	999.87	160.58	874.11	108.03	0.47	0.87	0.54
ANP, ng/day	67.36	21.02	18.73	5.41	75.72	30.03	66.27	29.10	0.23	0.29	0.40
Vasopressin, ng/day	2.48	0.98	2.10	1.11	2.25	1.14	1.59	0.14	0.47	0.76	0.85
PGEM, ng/day	14.66	0.82	11.36	0.49	14.66	1.29	14.75	0.38	0.063	0.051	0.051
ET-1, pg/day	6.92	0.26	9.30	0.90	9.62	1.16	**13.10***	**1.02**	**0.001**	**0.020**	0.380
H_2O_2, µmol/day	0.35	0.12	0.60	0.28	1.63	0.12	**3.06***	**0.84**	0.17	**0.021**	0.36
CH_2O, ml/h	n.d.		n.d.		n.d.		-3.65	0.32			
Creatinine Clearance, ml/min/100 g B.W.	n.d.		n.d.		n.d.		0.66	0.06			

Table S4: Plasma electrolytes, creatinine and hormones from rats on a high salt diet (HSD) or 1% NaCl water (HSW) for 7 days either an intramedullary vehicle infusion or class I HDAC inhibitor, MS275. * represents $P < 0.05$ from post hoc Dunnett's multiple comparison between vehicle and MS275.

	Vehicle		MS275		MS275		ANOVA
	HSD7				HSW7		
Sample size n =	4		5		5		
	mean	s.e.m.	mean	s.e.m.	mean	s.e.m.	P-value
Osmolality, mmol/Kg H_2O	299.00	1.78	305.80	4.49	293.80	2.92	0.07
Na^+, mmol/L	139.50	0.56	139.60	0.79	140.60	0.20	0.36
Cl^-, mmol/L	104.70	0.60	102.30	1.70	102.90	0.30	0.67
K^+, mmol/L	4.86	0.22	**3.86***	0.13	**4.01***	0.05	**0.0009**
Creatinine, mg/dL	0.47	0.03	0.41	0.01	**0.37***	0.02	**0.037**
Plasma renin conc, ng AngI /ml/h	6.75	0.25	6.93	0.17	6.22	0.23	0.08
Aldosterone, pg/ml	1941.00	321.20	1380.00	281.80	1227.00	164.90	0.18

Table S5: Metabolic cage data and excretion of control and *iHoxb7 HDAC1 knockout* (KO) mice on various salt diets.

	Normal Salt				High salt day 1				**High salt day 6**				Two Factor ANOVA		
Genotype *n* =	Control 16		*iHoxb7 Hdac1 KO 16*		Control 16		*iHoxb7 Hdac1 KO 16*		Control 16		*iHoxb7 Hdac1 KO 16*		P_{diet}	$P_{genotype}$	P_{DxG}
Mixed Sexes	mean	s.e.m.	mean	s.e.m.	mean	s.e.m.	mean	s.e.m.	mean	s.e.m.	mean	s.e.m.			
Food intake g/day	7.91	0.67	6.95	0.89	7.03	0.57	6.31	0.58	9.36	0.83	9.02	0.44	**<0.0001**	0.54	0.86
Water intake ml/day	1.65	0.17	1.43	0.26	7.92	0.70	7.94	1.18	16.45	1.45	16.08	1.97	**<0.0001**	0.80	0.79
UV ml/day	0.84	0.20	1.09	0.24	4.41	0.65	4.93	0.90	8.97	0.79	10.30	1.00	**<0.0001**	0.50	0.98
Genotype *n* =	Control 6		*iHoxb7 Hdac1 KO 7*		Control 6		*iHoxb7 Hdac1 KO 7*		Control 6		*iHoxb7 Hdac1 KO 7*				
UNaV mmol/day	0.21	0.16	0.37	0.23	1.95	0.28	1.94	0.50	5.03	0.68	5.12	0.28	**<0.0001**	0.89	0.84
UKV mmol/day	0.11	0.09	0.20	0.06	0.31	0.03	0.28	0.05	0.30	0.04	0.31	0.02	**0.01**	0.77	0.84
UNOx nmol/day	n.d.		n.d.		1211.00	235.60	1189.00	280.60	38.85	9.90	70.10	33.36	**<0.001**	0.97	0.88

Table S6: Plasma chemistries from control and *iHoxb7 Hdac1* knockout (KO) mice on various salt diets. * represents $P < 0.05$ from post hoc Sidak's multiple comparison between control and KO.

	Normal Salt				High salt day 6				Two Factor ANOVA		
Males *n* =	Control 5		*iHoxb7 Hdac1 KO 5*		Control 9		*iHoxb7 Hdac1 KO 3*		P_{diet}	$P_{genotype}$	P_{DxG}
	Mean	s.e.m	Mean	s.e.m	Mean	s.e.m	Mean	s.e.m			
Mass, g	29.5	2.1	28.0	2.6	27.5	1.8	26.0	1.7	0.38	0.50	0.99
PNa mmol/L	144.2	0.6	143.8	0.4	145.1	1.2	143.0	0.6	0.96	0.28	0.46
PK mmol/L	4.4	0.2	**3.7***	**0.2**	3.7	0.1	3.5	0.1	**0.015**	**0.037**	0.16
PCl mmol/L	110.8	1.5	112.4	1.1	114.9	1.1	114.3	0.3	**0.040**	0.71	0.44
BUN mg/dL	20.8	1.5	20.0	1.4	17.3	1.4	14.3	2.3	**0.019**	0.30	0.54
Hematocrit, %	36.4	0.8	37.2	1.0	34.6	1.1	34.3	2.4	0.10	0.84	0.71
HCO3 mg/dL	23.7	1.5	23.6	2.0	20.9	1.1	21.5	1.5	0.14	0.88	0.84

	Normal Salt				High salt day 6				Two Factor ANOVA		
Females *n* =	Control 4		*iHoxb7 Hdac1 KO 4*		Control 5		*iHoxb7 Hdac1 KO 6*		P_{diet}	$P_{genotype}$	P_{DxG}
	Mean	s.e.m	Mean	s.e.m	Mean	s.e.m	Mean	s.e.m			
Mass, g	21.2	0.5	19.0	1.2	21.4	0.8	19.4	0.7	0.69	**0.017**	0.92
PNa mmol/L	144.0	0.8	144.5	0.3	146.2	1.5	145.2	1.0	0.21	0.81	0.50
PK mmol/L	3.8	0.1	3.5	0.2	3.5	0.2	3.5	0.1	0.50	0.48	0.31
PCl mmol/L	114.7	0.7	110.0	0.7	111.4	1.5	113.3	1.5	0.98	0.35	**0.03**
BUN mg/dL	14.0	3.1	13.3	1.9	14.6	1.5	16.2	0.7	0.30	0.81	0.49
Hematocrit, %	37.0	1.1	37.3	0.8	38.2	0.6	38.2	0.6	0.95	0.24	0.36
HCO3 mg/dL	19.1	0.5	21.8	0.2	22.8	1.2	22.8	1.2	**0.01**	42.00	0.07

Table S7: Metabolic cage data and excretion of control and *iHoxb7 Hdac1/Hdac2 knockout* (*Hdac1/2KO*) mice on various salt diets.

* represents $P < 0.05$ from post hoc Sidak's multiple comparison between control and KO.

	**Low Salt**				**Normal Salt**				**High salt day 1**				**High salt day 6**				Two Factor ANOVA		
Males	control		*iHoxb7 Hdac1/2KO*		control		*iHoxb7 Hdac1/2KO*		control		*iHoxb7 Hdac1/2KO*		control		*iHoxb7 Hdac1/2KO*		P_{diet}	$P_{genotype}$	P_{DxG}
Sample size	14		13		14		13		14		13		14		13				
	mean	s.e.m.	mean	s.e.m.	mean	s.e.m.	mean	s.e.m.	mean	s.e.m.	mean	s.e.m.	mean	s.e.m.	mean	s.e.m.			
Food intake, g/day	7.78	0.65	9.79	0.52	10.64	0.51	10.77	0.36	9.92	0.84	9.36	0.75	10.54	0.46	10.54	0.50	**0.0033**	0.60	**0.03**
Sodium intake, mg/day	1.56	0.13	1.96	0.10	17.45	0.89	17.32	0.71	158.66	13.38	149.75	12.07	168.66	7.35	168.63	7.92	**<0.0001**	0.75	0.89
Water intake, ml/day	2.39	0.68	4.40	0.70	4.61	0.69	6.22	0.51	12.55	1.37	15.62	1.90	18.78	0.94	**24.88***	**2.21**	**<0.0001**	**0.012**	0.19
Urine Flow , ml/day	1.22	0.14	**3.33***	**0.53**	2.06	0.32	2.86	0.34	8.91	1.03	11.50	1.42	13.27	0.84	**18.76***	**1.44**	**<0.0001**	**0.005**	0.03
UNaV, mmol/day	0.13	0.03	0.19	0.04	n.d.		n.d.		3.15	0.34	3.48	0.46	5.08	0.23	5.25	0.40	**<0.0001**	0.49	0.86
UKV, mmol/day	0.38	0.06	0.44	0.07	n.d.		n.d.		0.69	0.12	0.70	0.12	0.47	0.04	0.41	0.029	**0.0007**	0.96	0.63
Vasopressin, pg/day n= 8-9	844.00	263.20	578.40	137.33	n.d.		n.d.		3317.00	896.64	2140.10	602.20	2391.87	1932.60	414.90	338.45	**0.01**	0.180	0.45

	**Low Salt**				**Normal Salt**				**High salt day 1**				**High salt day 6**				Two Factor ANOVA		
Females	control		*iHoxb7 Hdac1/2KO*		control		*iHoxb7 Hdac1/2KO*		control		*iHoxb7 Hdac1/2KO*		control		*iHoxb7 Hdac1/2KO*		P_{diet}	$P_{genotype}$	P_{DxG}
Sample size	21		16		21		16		21		16		21		16				
	mean	s.e.m.	mean	s.e.m.	mean	s.e.m.	mean	s.e.m.	mean	s.e.m.	mean	s.e.m.	mean	s.e.m.	mean	s.e.m.			
Food intake, g/day	9.01	0.59	8.60	0.62	10.10	0.53	10.67	0.72	8.21	0.49	8.50	0.71	10.34	0.53	10.54	0.84	**<0.0001**	0.780	0.780
Sodium intake, mg/day	1.80	0.12	1.72	0.12	16.16	0.84	17.08	1.15	131.30	7.85	135.99	11.36	165.46	8.47	168.63	13.38	**<0.0001**	0.72	0.98
Water intake, ml/day	2.35	0.34	3.68	0.65	4.99	0.61	5.09	0.74	9.03	0.91	12.03	1.05	17.87	0.86	23.34	2.51	**<0.0001**	0.78	0.79
Urine Flow , ml/day	1.73	0.22	2.36	0.39	2.85	0.32	3.22	0.39	5.76	0.79	**8.15***	**0.95**	11.03	0.87	**14.57***	**1.30**	**<0.0001**	**0.029**	**0.020**
UNaV, mmol/day	0.17	0.038	0.15	0.018	n.d.		n.d.		2.24	0.31	1.9	0.19	4.05	.25	4.2	0.16	**<0.0001**	0.69	0.43
UKV, mmol/day	0.21	0.03	0.21	0.035	n.d.		n.d.		0.33	0.052	0.273	0.028	0.266	0.016	0.26	0.014	**0.01**	0.48	0.57
Vasopressin, pg/day	398.25	63.7	392.69	70.65	n.d.		n.d.		1129.42	204.3	1747.7	398.64	2139.4	179.41	2467.22	467.45	**<0.0001**	0.26	0.32

n.d. – not determined.

Table S8: Plasma chemistries of control and *iHoxb7 Hdac1/Hdac2 knockout* (*Hdac1/2KO*) mice on various salt diets.

	Normal Salt				**High salt day 6**				Two Factor ANOVA		
Males *n* =	Control 9		*iHoxb7 Hdac1/2KO 7*		Control 13		iHoxb7 Hdac1/2KO 10		P_{diet}	$P_{genotype}$	P_{DxG}
	Mean	s.e.m	Mean	s.e.m	Mean	s.e.m	Mean	s.e.m			
PNa mmol/L	143.4	0.4	142.7	0.3	144.6	0.3	143.7	0.4	**0.0075**	**0.037**	0.81
PK mmol/L	4.1	0.1	4.1	0.1	3.9	0.1	3.89	0.1	0.077	0.74	0.94
PCl mmol/L	111.6	0.4	112.1	0.6	110.5	0.7	110.9	0.6	0.079	0.43	0.90
BUN mg/dL	25.4	1.4	30.3	3.1	19.5	0.7	22.4	1.2	0.69	**0.040**	0.76
Hematocrit, %	35.0	0.4	35.2	0.8	35.6	0.6	35.9	0.8	0.39	0.69	0.99
HCO3 mg/dL	20.9	0.6	22.1	0.3	23.9	0.5	23.58	0.6	0.0005	0.44	0.20

	Normal Salt				**High salt day 6**				Two Factor ANOVA		
Females *n* =	Control 10		iHoxb7 Hdac1/2KO 6		Control 7		iHoxb7 Hdac1/2KO 6		P_{diet}	$P_{genotype}$	P_{DxG}
	Mean	s.e.m	Mean	s.e.m	Mean	s.e.m	Mean	s.e.m			
PNa mmol/L	142.0	0.4	141.5	0.3	142.0	0.4	142.7	0.4	0.13	0.93	0.32
PK mmol/L	3.6	0.2	3.4	0.2	3.3	0.1	3.4	0.1	0.47	0.96	0.47
PCl mmol/L	111.3	0.8	112.3	0.3	110.7	0.6	112.9	0.9	0.29	0.29	0.39
BUN mg/dL	23.3	1.0	26.2	4.4	19.3	0.7	24.7	1.1	0.18	**0.04**	0.53
Hematocrit, %	34.7	1.0	33.7	1.2	33.0	0.5	34.2	0.7	0.53	0.93	0.25
HCO3 mg/dL	21.5	1.0	22.0	1.4	20.3	1.0	22.0	0.3	0.56	0.3	0.56

Table S9: Markers used to determine identity of cluster populations from the snRNA-Seq.

See excel file

Table S10: Differentially expressed genes in each cluster

See excel file

Table S11: Primer sequences used in the study.

Target	Primer name	5' to 3'	Function	expected band	size of band bp
Hdac1	*Hdac1 Forward*	CTGCCTCTGCTTCCTTA	genotyping	WT	228
	Hdac1 Reverse	GTCCGTCTGCTGCTTAT	genotyping	Floxed	328
	Hdac1 recombinant primer	GTTACTGTACTGTGAGCAAAAGG	recombination	Mutant	450
Hdac2	*Hdac2 Forward*	ATTCAAAGGCAGCAGCAGGAGA	genotyping	WT	384
	Hdac2 Reverse	GTCAGCTAGTAGTGCTTCTTGG	genotyping	Floxed	290
	Hdac2 recombinant primer	GTCTCTGAGTAAAAAGACACAAGC	recombination	Mutant	450
Hoxb7-rtTA	*Hoxb7F1*	GGTCACGTGGTCAGAAGAGG	genotyping	positive	700
	Hoxb7R2	CTCCAGGCCACATATGATTAG	genotyping		
Lc-1	*Lc1F1*	TGCCTGCATTACCGGTCGATGC	genotyping	positive	417
	Lc1R1	CCATGAGTGAACGAACCTGGTCG	genotyping		
Pax8-rtTA	*Pax8F1*	CCATGTCTAGACTGGACAAGA	genotyping	positive	596
	Pax8R1	CTCCAGGCCACATATGATTAG	genotyping		
Internal control primers	*Forward*	CTAGGCCACAGAATTGAAAGATCT	genotyping	positive	324
	Reverse	GTAGGTGGAAATTCTAGCATCATCC	genotyping		

Table S12: Antibodies used in the study.

Antibody	Sequence	Host	Concentration or dilution of stock provided	Company	Cat #	Lot # or clone #	Location
**Westerns**							
ace-H3	human	rabbit polyclonal	1mg/ml	EMD millipore	06-599	2724352	Billerica, MA
β-actin	?	mouse monoclonal	1/50,000	Sigma	A1978	087M4880V	St. Louis, MO
AQP2	Human	goat polyclonal	2 µg/10ml	Santa Cruz	sc-9882	F413	Santa Cruz, CA
HDAC1	human	goat polyclonal	2 µg/10ml	Santa Cruz	sc-6298	K0413	Santa Cruz, CA
HDAC2	human	mouse monoclonal	1/1000	Cell Signaling	5113	3F3	Danvers, MA
HDAC3	human	mouse monoclonal	1/1000	Cell Signaling	3949	7G6C5	Danvers, MA
HDAC8	human	rabbit polyclonal	2 µg/10ml	Santa Cruz	sc-11405	612	Santa Cruz, CA
NOS1	rat	rabbit polyclonal	2 µg/10ml	Santa Cruz	sc-648	B2613	Santa Cruz, CA
NOS2	human	mouse monoclonal	2ug/10ml	Santa Cruz	sc-7271	M19	Santa Cruz, CA
NOS3	human	mouse monoclonal	2.5 µg/10ml	BD biosciences	610296	clone 3	Franklin Lakes, NJ
PAQP2 261	rat	rabbit polyclonal	1/1000	PhosphoSolutions	p112-261	cs112b	Aurora, CO
pNOS3-1177	human	rabbit monoclonal	1/1000	Cell Signaling	9570	C9C3	Danvers, MA
pNOS3-495	human	rabbit polyclonal	1/1000	Cell Signaling	9574	lot 2	Danvers, MA
Total-H3	human	rabbit polyclonal	1mg/ml	EMD millipore	05-928	2603378	Billerica, MA
**Immunohistochemistry**							
HDAC1	human	rabbit monoclonal	1/5000	Abcam	AB109411	GR53419-13	Cambridge, UK
HDAC2	human	rabbit monoclonal	1/1000	Abcam	AB32117	GR112991-15	Cambridge, UK

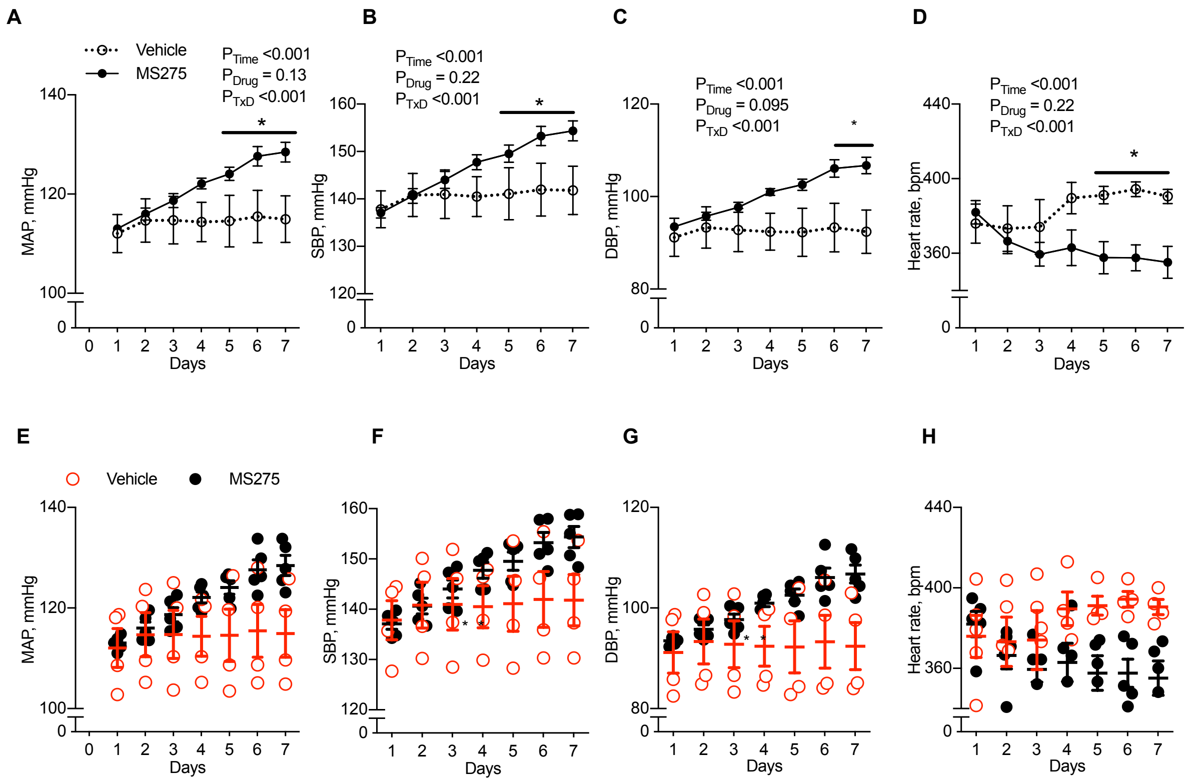

Fig S1. Intramedullary infusion of the class I HDAC inhibitor, MS275 (closed symbols), resulted in a significant increase in mean arterial pressure (MAP) and decrease in heart rate compared vehicle infused rats (open symbols), in rats fed 7 days of high salt diet. (*A*) MAP (*B*) systolic blood pressure (SBP), (*C*) diastolic blood pressure (DBP), and (*D)* Heart rate. *n* = 4-5, Two Factor ANOVA, * Significant difference from vehicle $P < 0.05$. E-H) individual animals plotted for vehicle treated (red) and MS275 (black), mean +/- s.e.m.

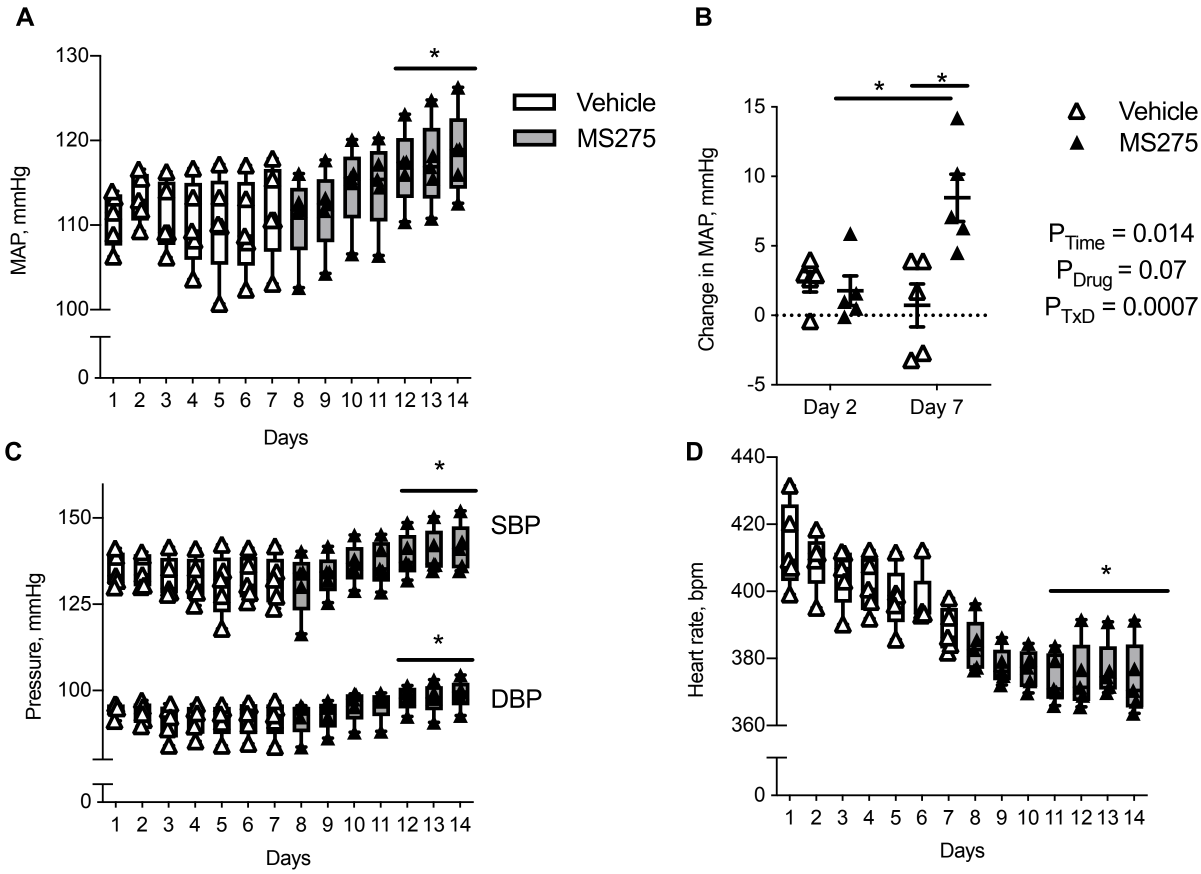

Fig S2. On day 1-14 rats were salt loaded with 1% NaCl water provided *ad libitium*. Intramedullary infusion of the Class I HDAC inhibitor, MS275, starting on day 7 resulted in an increase in (***A***) mean arterial pressure (MAP). *P<0.05 compared to vehicle day 7, paired, two-tailed Student's t-test. (**B**) The change in MAP from day 1 of vehicle or MS275 infusion and day 2 or day 7 is plotted. MS275 infusion lead to a significant increase in MAP (*n* = 5/ group, Two-Way, repeated measures ANOVA, *P< 0.05). (***C***) systolic blood pressure (SBP) and diastolic blood pressure (DBP). (**D**) heart rate.). *P<0.05 compared to vehicle day 7. Box plots from minimum to maximum with individual points plotted.

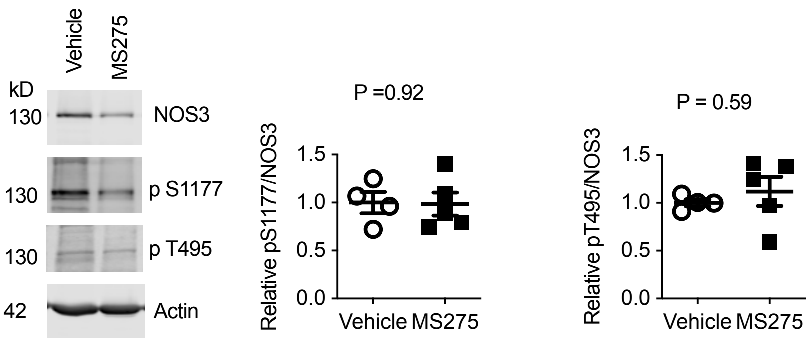

Fig S3: Inner medullary NOS3 phosphorylation status from vehicle and MS275 intramedullary infused rats on a high salt pellet diet. Although there was a tendency for MS275 to reduce NOS3 phosphorylation, it was in proportion to the amount of total NOS3. Unpaired, two tailed, Student's *t*-test.

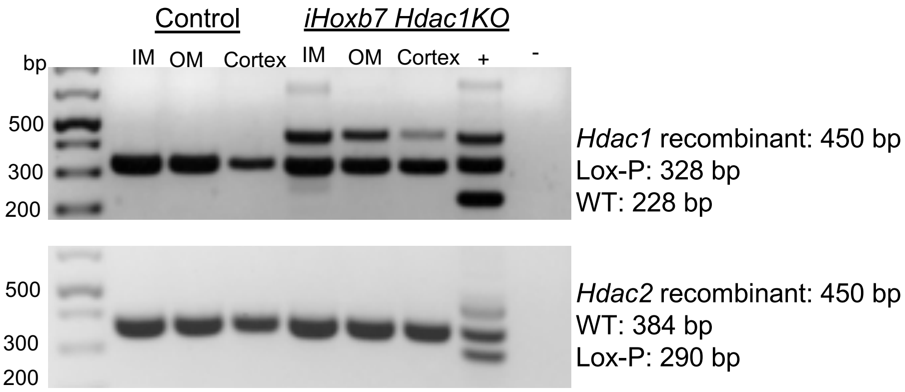

Fig S4. Confirmation of *Hdac1* genetic recombination and knockout in the collecting duct. PCR of DNA from a control or *iHoxb7 Hdac1KO* mouse. Recombinant DNA was found in the inner medulla (IM), outer medulla (OM), and cortex of the *iHoxb7 Hdac1KO* mouse. *Hdac2* primers were used also to demonstrate only wild type (WT) *Hdac2* is expressed in this colony (no recombination). Positive control (+) was from a *iHoxb7 Hdac1/2KO* mouse, and negative control (-) was water.

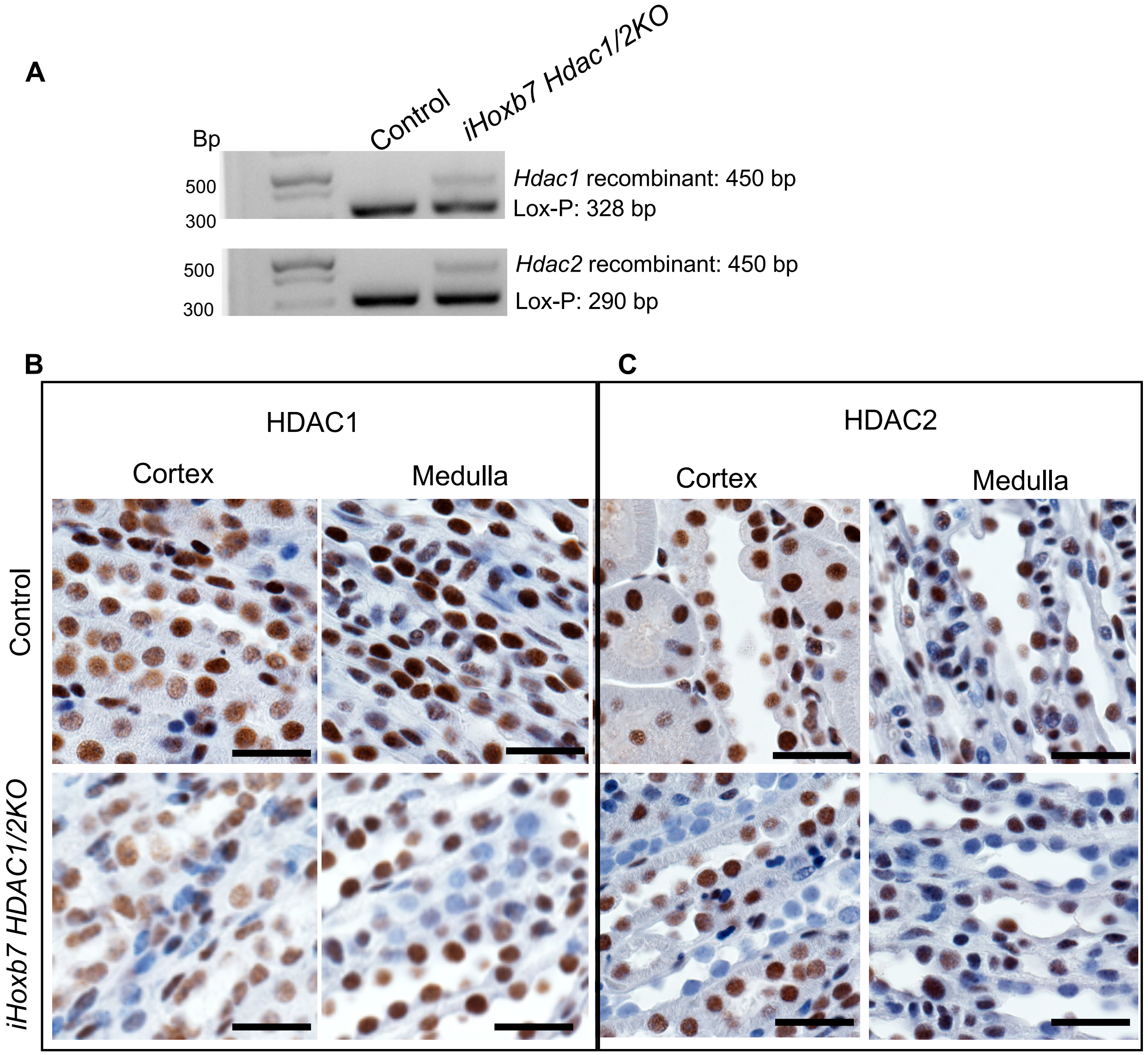

Fig S5: Confirmation of *Hdac1* and *Hdac2* genetic recombination and knockout in the collecting duct. **A**) PCR of DNA from a control or *iHoxb7 Hdac1/2KO* mouse. Recombinant bands were found in only the predicted knockout mice. **B**) Representative immunohistochemistry of kidney sections from control and *iHoxb7 Hdac1/2KO* mice. HDAC1 (brown staining) is expressed in the nuclei of the kidney epithelium in control mice, but absent in knockout mice (hematoxylin stained blue nuclei). **C**) HDAC2 is expressed in the CCD and IMCD of control mice, but is absent in *iHoxb7 Hdac1/2KO* mice. Scale bar = 20 microns.

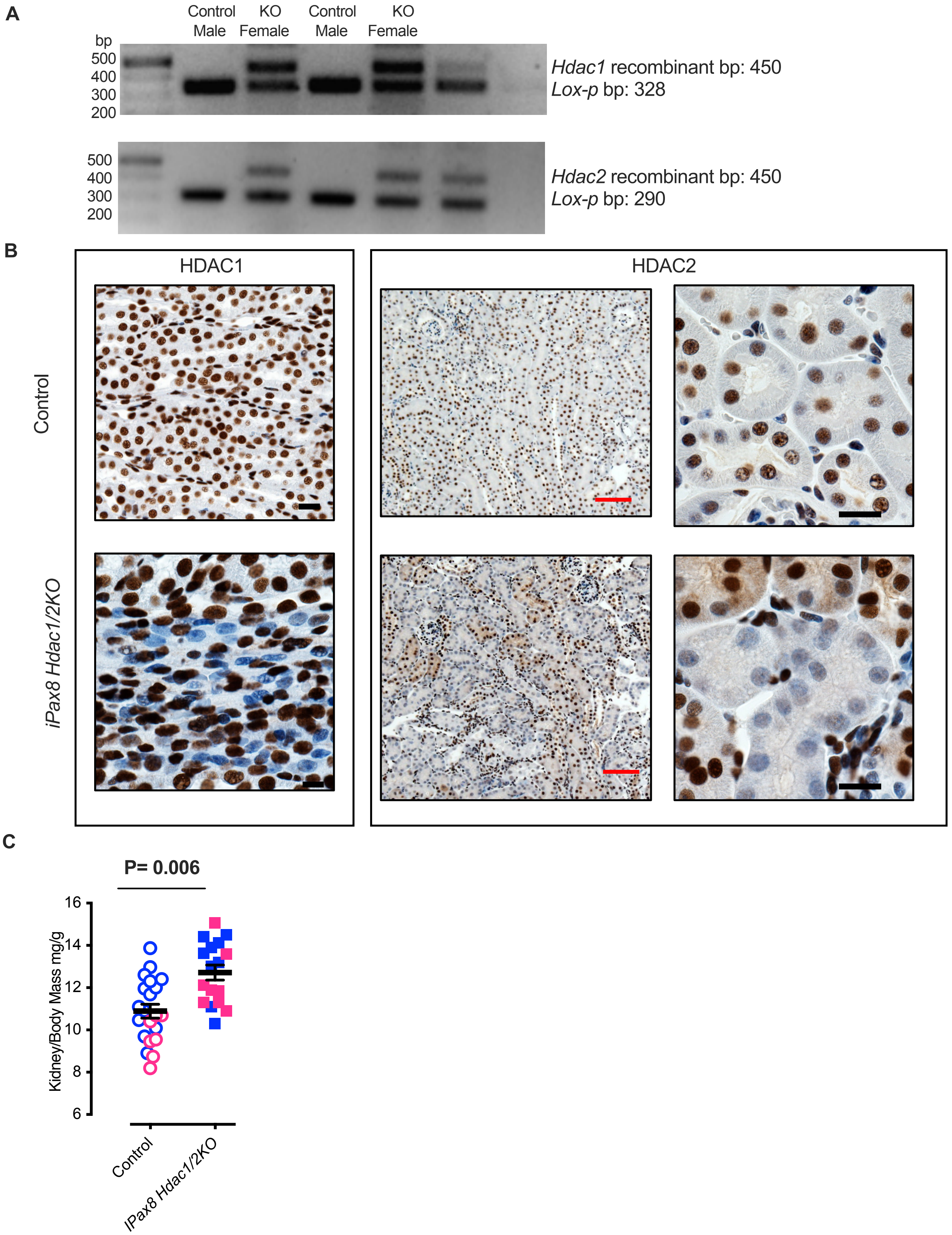
A
Control Male
KO Female
Control Male
KO Female
bp
500
400
300
200
Hdac1 recombinant bp: 450
Lox-p bp: 328
500
400
300
200
Hdac2 recombinant bp: 450
Lox-p bp: 290
B
HDAC1
HDAC2
Control
iPax8 Hdac1/2KO
C
P= 0.006
Kidney/Body Mass mg/g
16
14
12
10
8
6
Control
iPax8 Hdac1/2KO

Fig S6. Confirmation of *Hdac1 and Hdac2* genetic recombination and knockout in the nephron epithelium (*iPax8 Hdac1/Hdac2*KO = *iPax8Hdac1/2KO*). **A**) PCR of DNA from a control or *iPax8Hdac1/2KO* mouse. Recombinant bands were found in only the predicted knockout male and female mice. **B**) Representative immunohistochemistry of kidney sections from control and *iPax8Hdac1/2KO* mice. HDAC1 (brown staining) is expressed in the nuclei of the kidney epithelium in control mice, but absent in knockout mice (hematoxylin stained blue nuclei). Black scale bar = 20 microns, red scale bar = 100 microns. (**C**) Knockouts also have a greater kidney to body mass ratio. P value from two tailed, unpaired, Student's *t*-test reported.

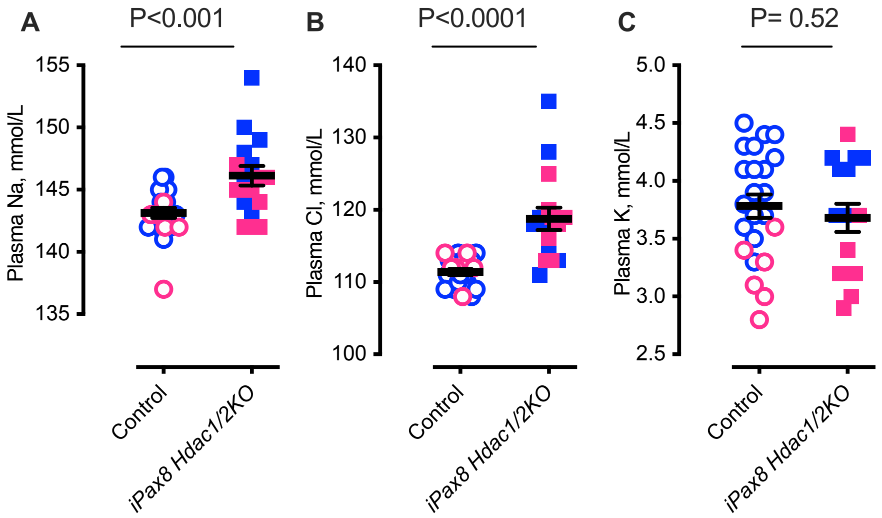

Fig S7. Individual reports of plasma electrolytes and kidney to body mass ratio in male (blue) and female (pink) control and *iPax8 Hdac1/Hdac2* knockout (*iPax8Hdac1/2KO*) mice. (**A**) sodium, (**B**) chloride, (**C**) potassium. Results of unpaired, two-tailed, Student's *t*-test reported.

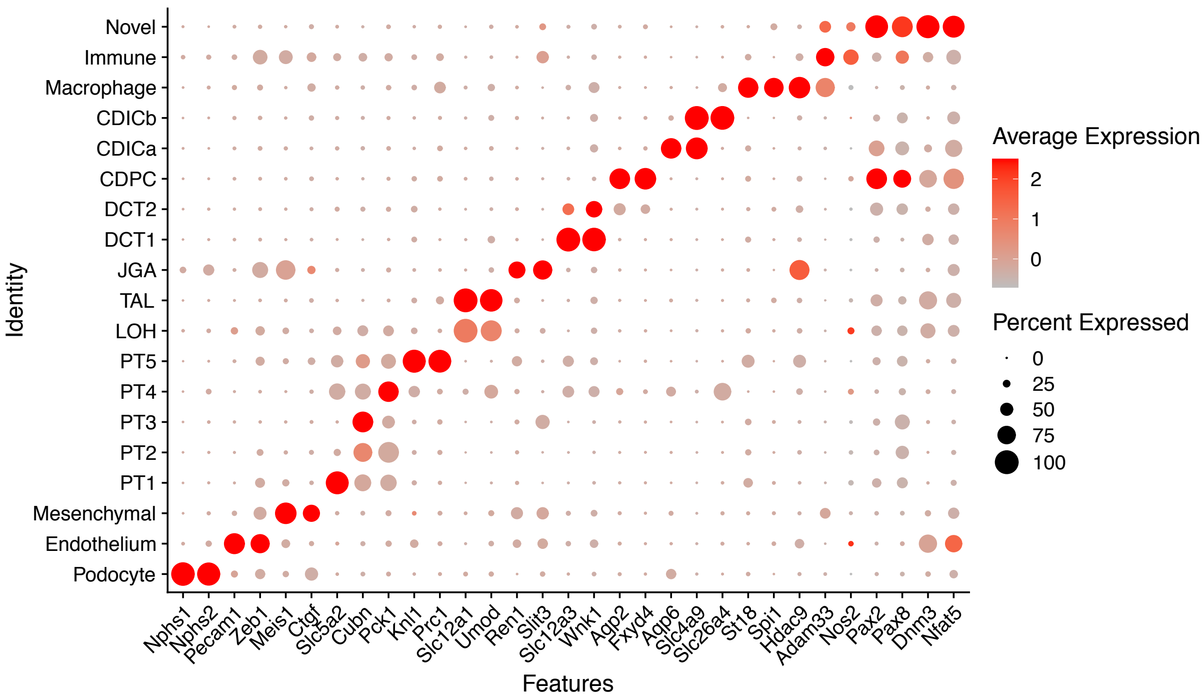

Fig S8. Dotplot of conserved genes among the 19 cell types identified in our snRNA-Seq integrated data set. Average expression represents standard deviation compared to all nuclei (e.g. 2 represents 2 standard deviations above the whole dataset).

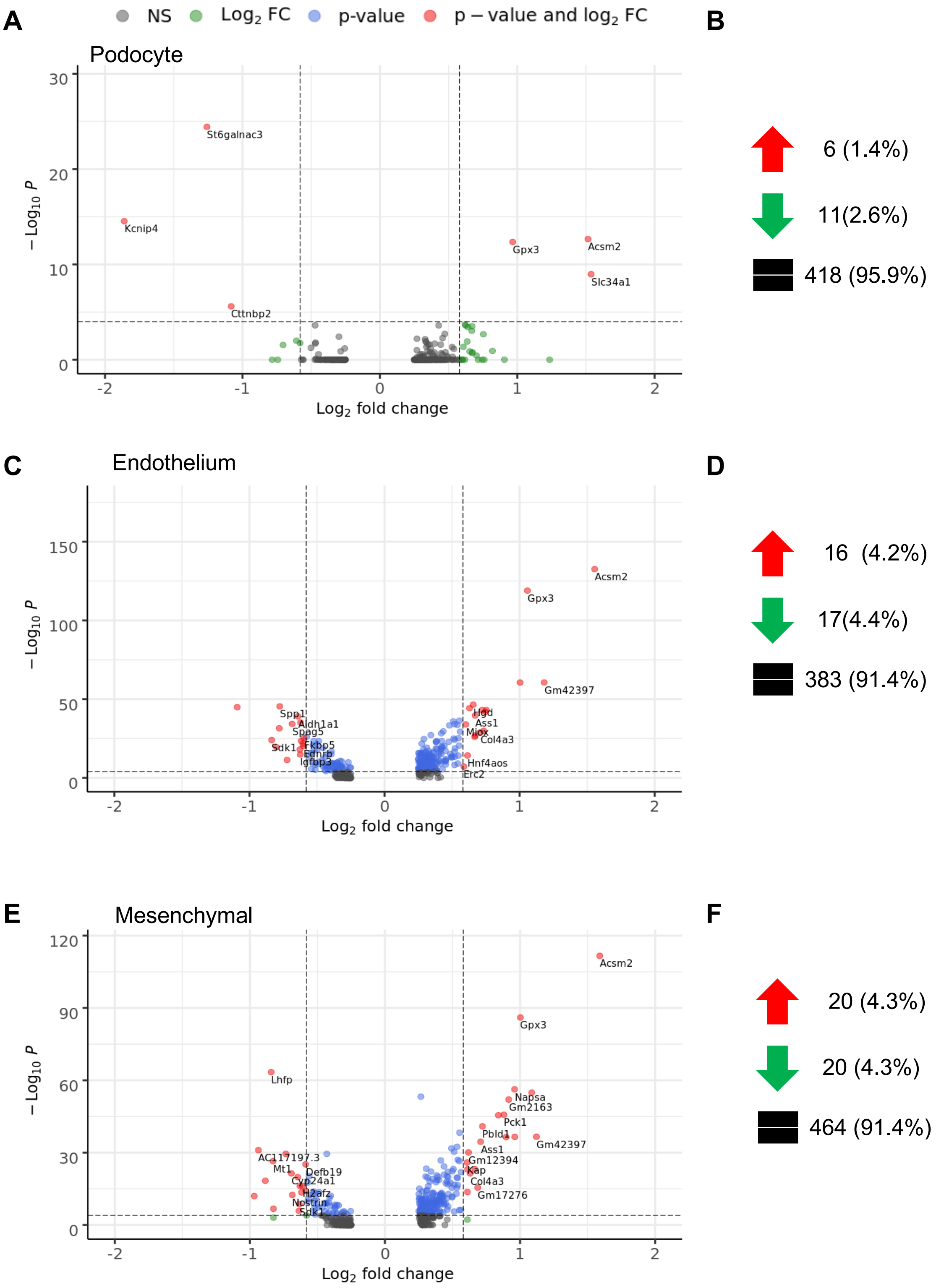

Fig. S9. Volcano plots of the differentially expressed genes in each cluster. (**A, C, E**) The average log fold change (Log_2FC) is of control *iPax8 Hdac1/Hdac2*KO. The dashed vertical lines represent a 1.5 fold increase or decrease in expression, and the dashed horizontal lines represent an adjusted p value greater than 10^{-5}. Dots

to the right represent greater expression in control, and dots to the left represent greater expression in the knockout mice. Grey represents genes that are not statistically significant, green represents log2FC > 0.58, blue represents $p < 10^{-5}$, and red represents genes that are both log2FC > 0.58 and $p < 10^{-5}$. (**B, D, F)** Summary of all differentially expressed genes that were significantly increased (red arrow) or decreased (green arrow) in the *iPax8 Hdac1/Hdac2KO* mice ($p < 0.05$), or unchanged (=). Count (%) reported for each cluster. (**A, B)** podocyte (cluster 1), (**C, D**) endothelium (cluster 2), (**E, F**) mesenchymal (cluster 3).

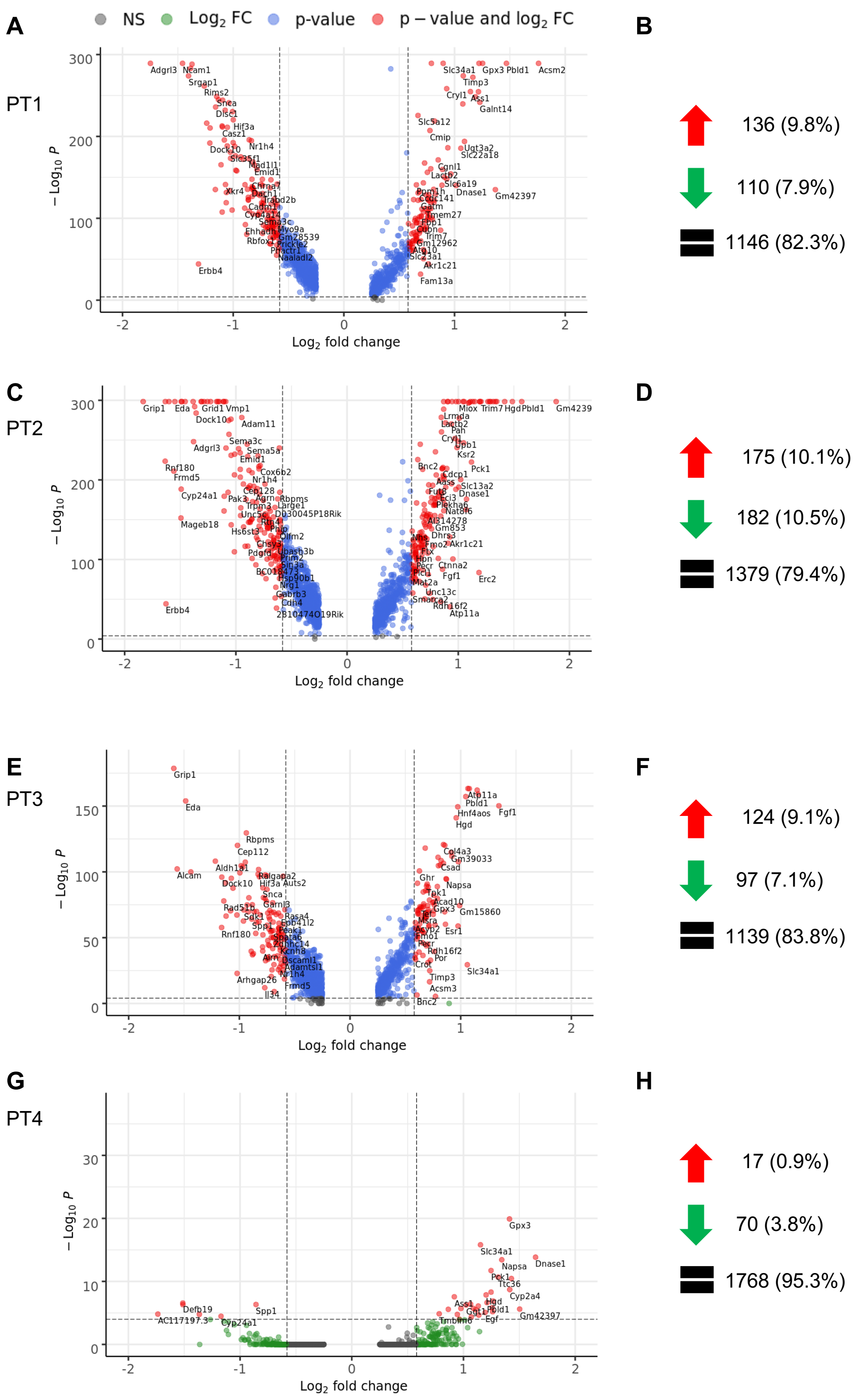

Fig. S10. Volcano plots of the differentially expressed genes in the proximal tubules (PT). (**A, B**) PT1 (cluster 4), (**C, D**) PT2 (cluster 5), (**E, F**) PT3 (cluster 6), (**G, H**) PT4 (cluster 7).

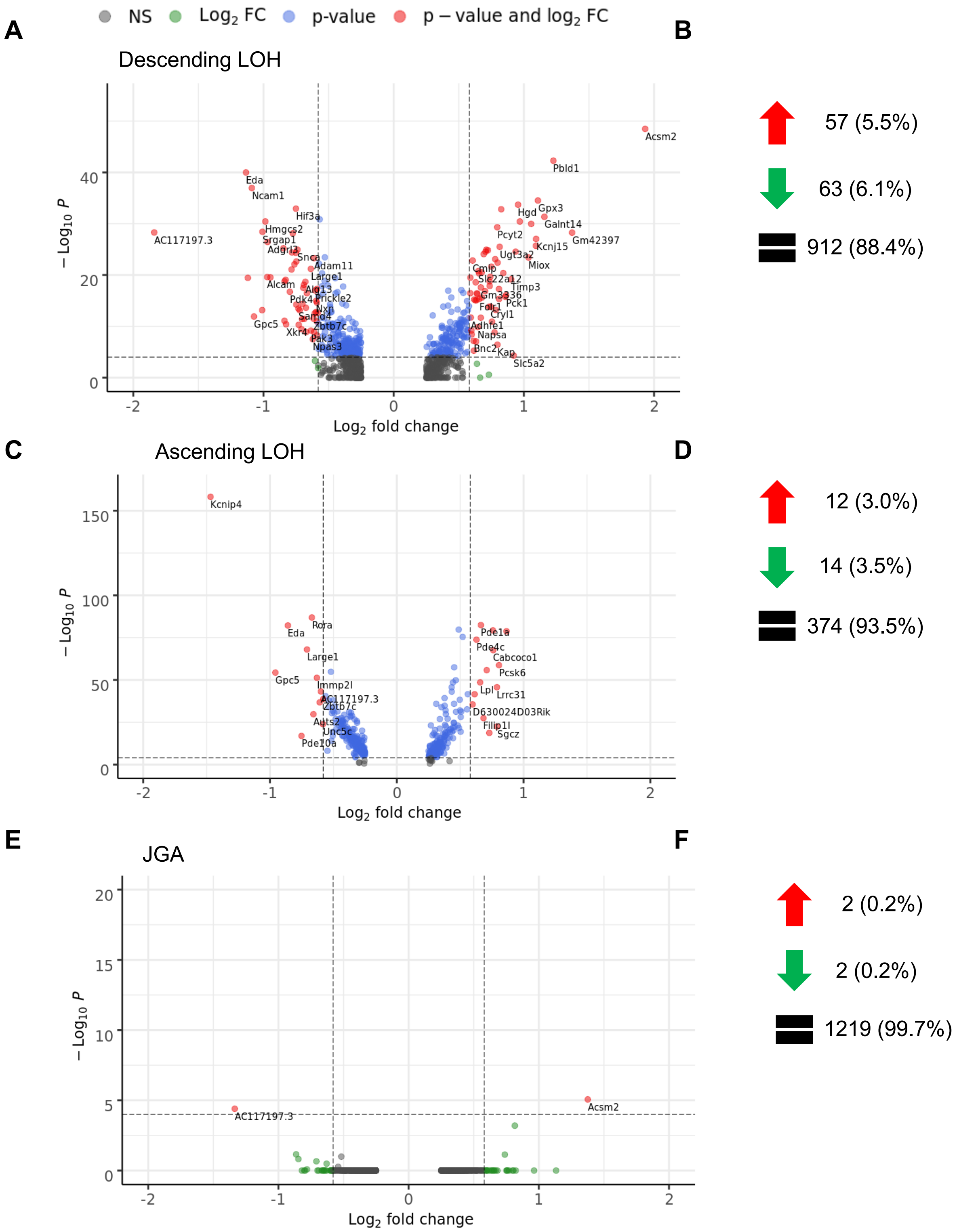

Fig. S11. Volcano plots of the differentially expressed genes in the loop of Henle (LOH) and juxtaglomerular apparatus (JGA). (**A, B)** descending LOH (cluster 9), (**C, D**) ascending LOH (cluster 10), (**E, F**) JGA (cluster 11).

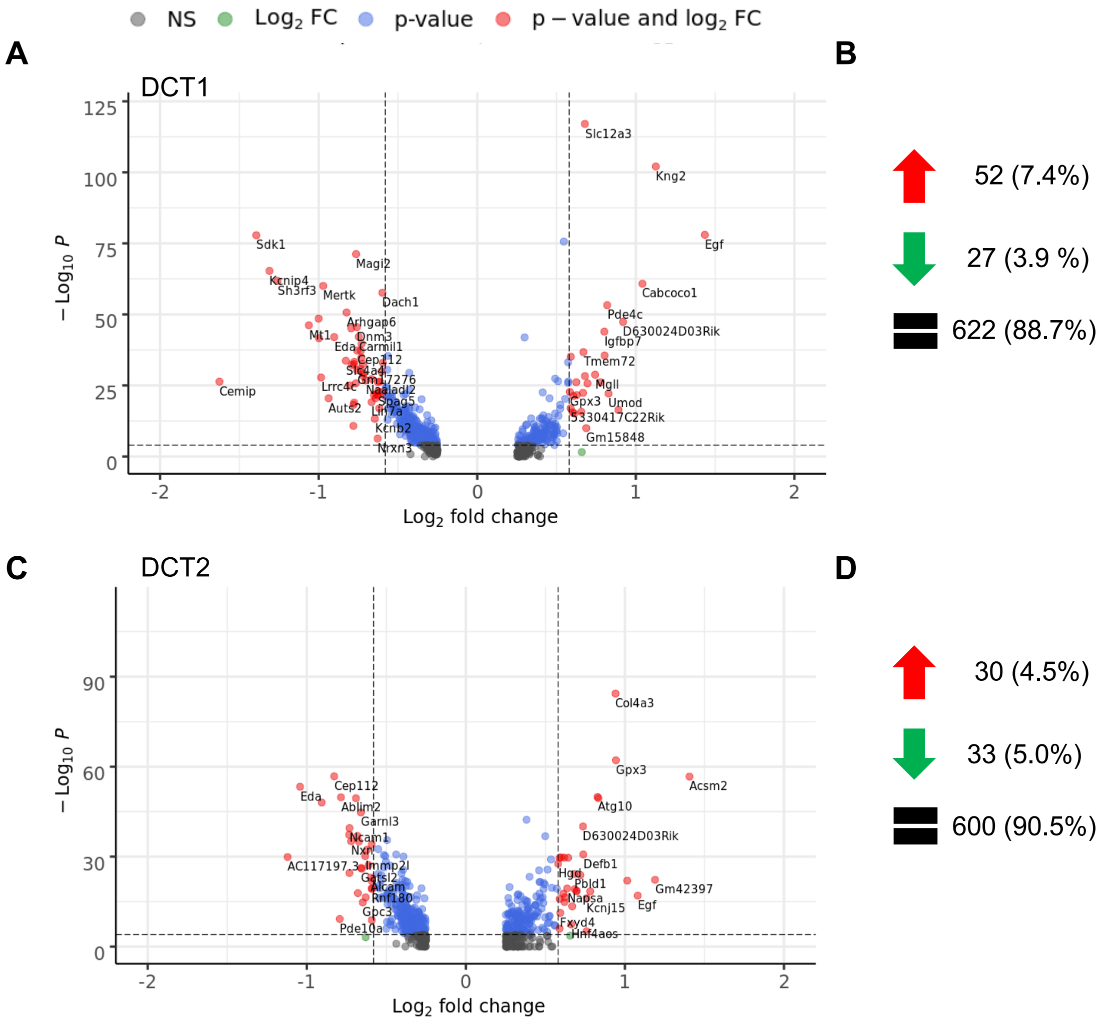

Fig. S12. Volcano plots of the differentially expressed genes in the distal convoluted tubule (DCT). (**A, B)** DCT1 (cluster 12), (**C, D**) DCT2 (cluster 13).

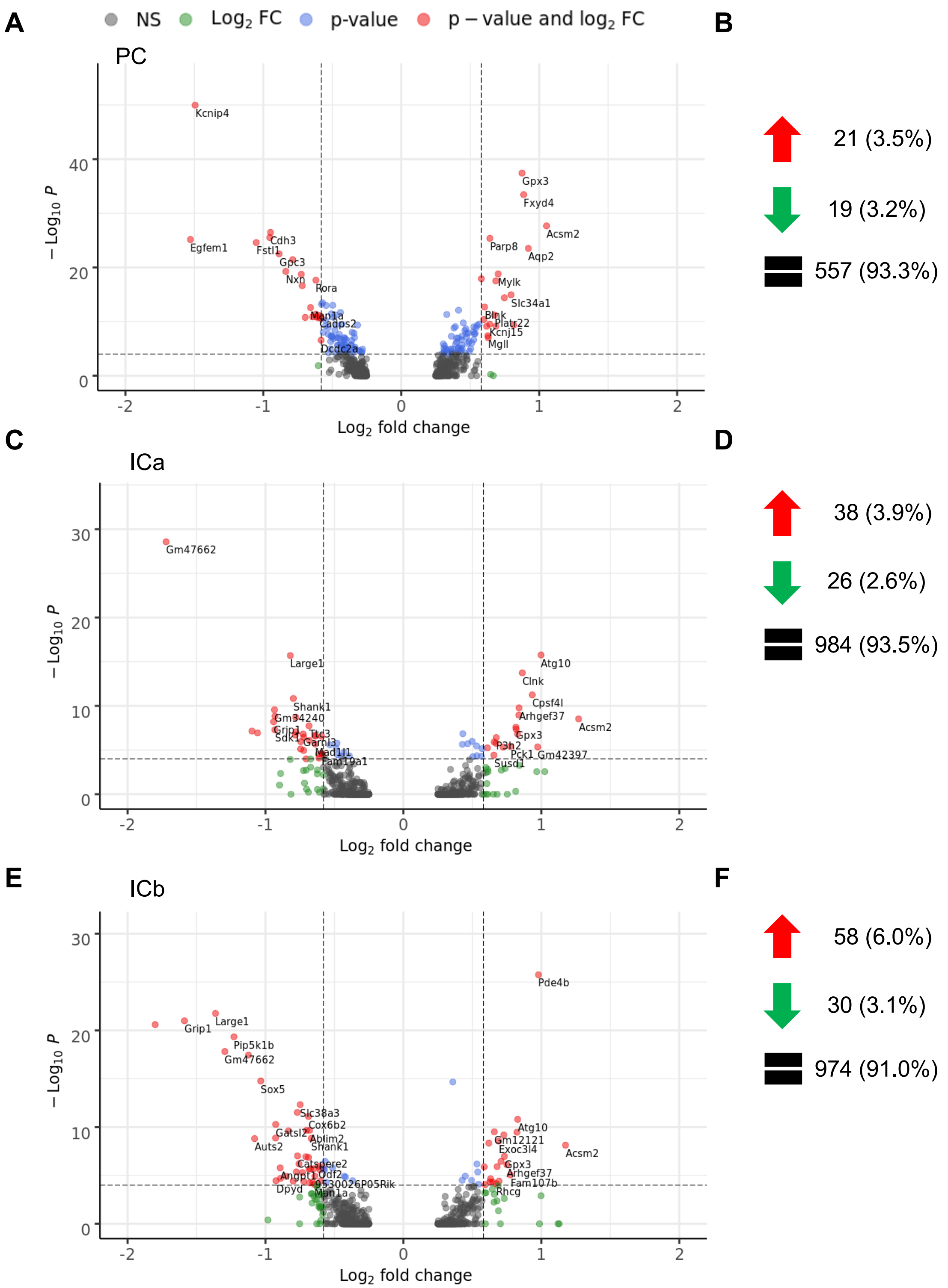

Fig. S13. Volcano plots of the differentially expressed genes in the collecting duct. (**A, B)** Principal cell (PC, cluster 14), (**C, D**) intercalated cell, subtype alpha (ICa) (cluster 15). (**E, F**) intercalated cell, subtype beta (ICb) (cluster 16).

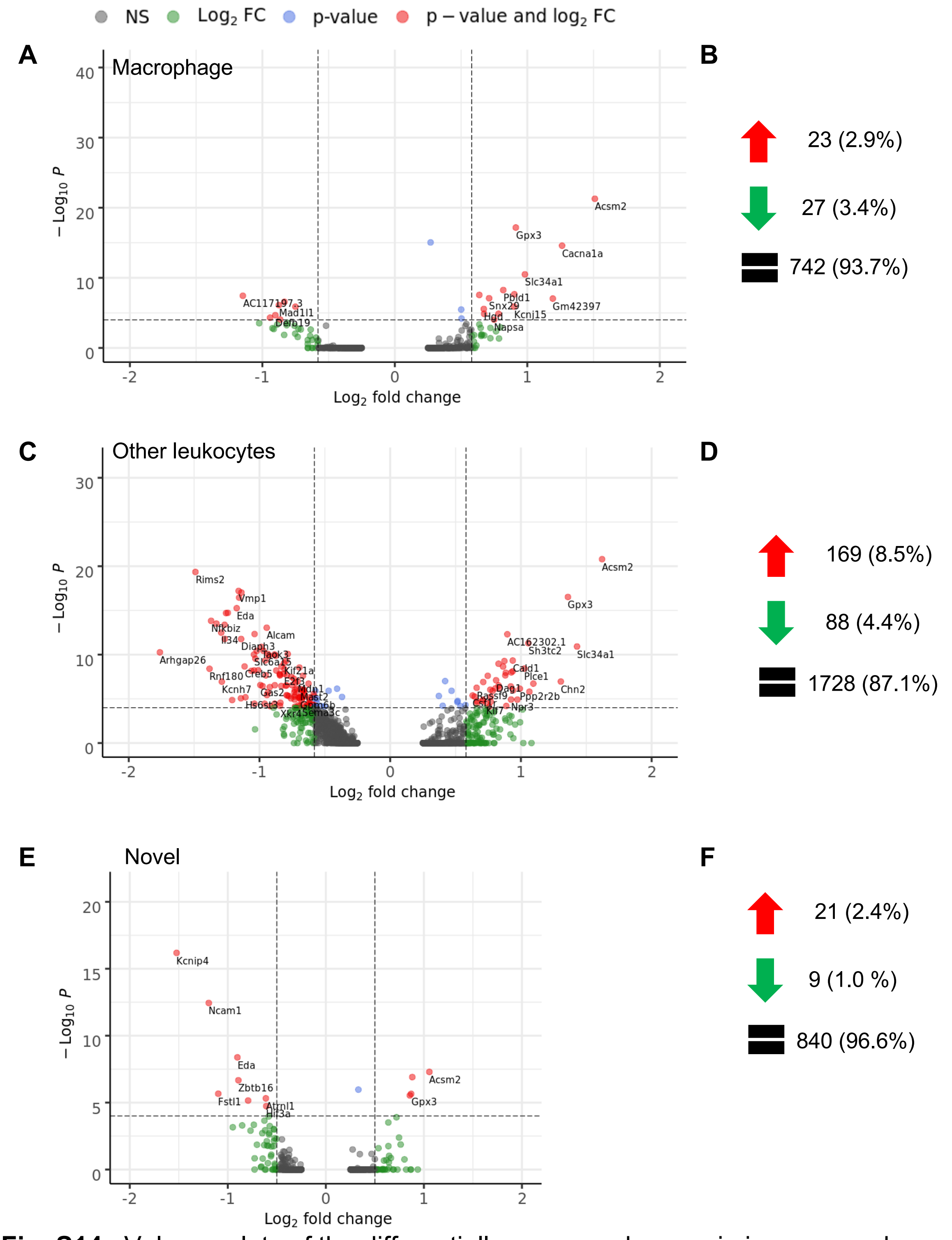

Fig. S14. Volcano plots of the differentially expressed genes in immune and novel cells. (**A, B)** Macrophages (cluster 17) (**C, D**) other leukocytes (cluster 18). (**E, F**) Novel cells (cluster 19).

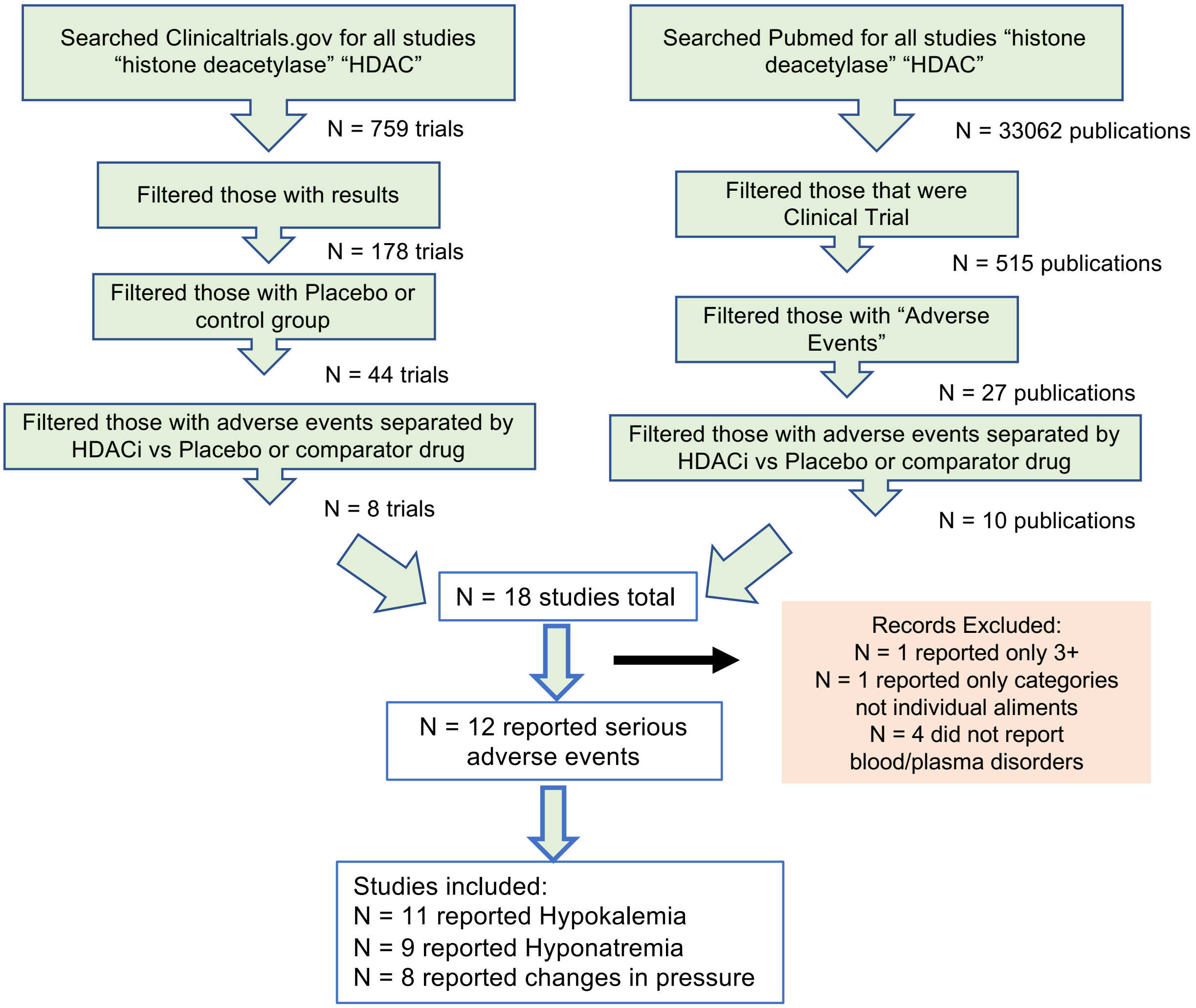

4/12 studies reported polyuria, urine inconsistency, or urine retention.

Fig S15: Work flow for the systematic review used in the metanalysis.

A. Any Fluid-electrolyte Issue

Study	Experimental Events	Experimental Total	Control Events	Control Total	OR	95%-CI	Weight (fixed)	Weight (random)
San-Miguel et al. 2016	224	381	156	377	2.02	[1.51; 2.70]	64.9%	23.5%
NCT01802333	20	381	0	377	42.81	[2.58; 710.52]	0.0%	2.3%
Kim et al. 2018	2	186	4	184	0.49	[0.09; 2.70]	4.0%	5.5%
NCT00473889	5	124	3	124	1.69	[0.40; 7.25]	2.9%	7.0%
NCT00481078	10	63	1	32	5.85	[0.71; 47.90]	1.1%	3.9%
Krug et al. 2015	5	329	1	329	5.06	[0.59; 43.56]	1.0%	3.8%
Prebet et al. 2014	36	98	13	99	3.84	[1.88; 7.84]	8.2%	15.8%
Witta et al. 2012	8	65	2	63	4.28	[0.87; 21.01]	1.8%	6.1%
Norsworthy et al. 2016	2	26	1	12	0.92	[0.07; 11.22]	1.3%	2.9%
Ramalingam et al. 2010	32	62	7	32	3.81	[1.44; 10.10]	4.5%	11.7%
Jiang et al. 2019	34	244	0	121	39.83	[2.42; 655.42]	0.0%	2.4%
NCT00773747	20	315	11	320	1.90	[0.90; 4.04]	10.3%	15.1%
**Fixed effect model**		**2274**		**2070**	**2.69**	**[2.16; 3.37]**	**100.0%**	**--**
**Random effects model**					**2.78**	**[1.77; 4.36]**	**--**	**100.0%**

Heterogeneity: $I^2 = 43\%$, $\tau^2 = 0.2043$, $p = 0.05$

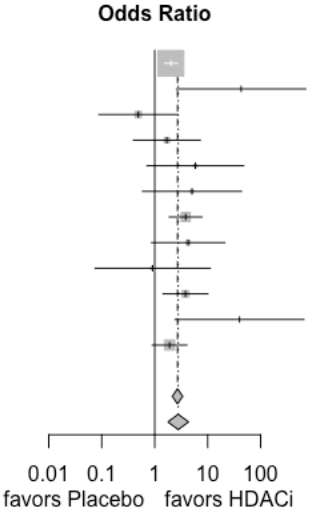

B. Hyponatremia

Study	Experimental Events	Experimental Total	Control Events	Control Total	OR	95%-CI	Weight (fixed)	Weight (random)
San-Miguel et al. 2016	25	381	18	377	1.40	[0.75; 2.61]	68.2%	57.6%
NCT01802333	2	381	0	377	4.97	[0.24; 103.94]	0.0%	2.4%
NCT00473889	2	124	2	124	1.00	[0.14; 7.21]	7.9%	5.7%
NCT00481078	3	63	0	32	3.76	[0.19; 75.05]	0.0%	2.5%
Krug et al. 2015	4	329	1	329	4.04	[0.45; 36.31]	4.0%	4.6%
Prebet et al. 2014	12	98	2	99	6.77	[1.47; 31.09]	7.0%	9.6%
Norsworthy et al. 2016	2	24	0	26	5.89	[0.27; 129.15]	0.0%	2.3%
Ramalingam et al. 2010	12	62	3	32	2.32	[0.60; 8.91]	12.9%	12.4%
Jiang et al. 2019	7	244	0	121	7.67	[0.43; 135.48]	0.0%	2.7%
**Fixed effect model**		**1706**		**1517**	**2.40**	**[1.50; 3.84]**	**100.0%**	**--**
**Random effects model**					**2.05**	**[1.28; 3.29]**	**--**	**100.0%**

Heterogeneity: $I^2 = 0\%$, $\tau^2 = 0$, $p = 0.55$

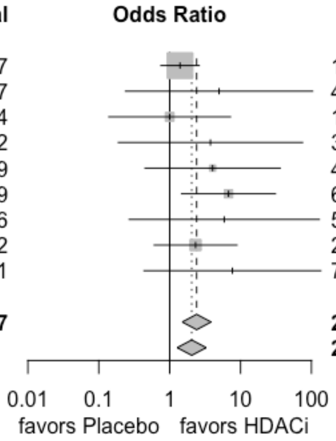

Fig S16: Forest plots of odds ratios (ORs) for datasets related to placebo/standard of care versus histone deacetylase inhibitor (HDACi). (**A**) Any fluid-electrolyte issue (see Supporting information for definition), and (**B**) hyponatremia.

A. Hypokalemia

Study	Experimental Events	Experimental Total	Control Events	Control Total	OR	95%-CI	Weight (fixed)	Weight (random)
San-Miguel et al. 2016	8	381	4	377	2.00	[0.60; 6.70]	20.4%	17.5%
NCT01802333	6	381	0	377	13.07	[0.73; 232.82]	0.0%	3.1%
Kim et al. 2018	1	186	0	184	2.98	[0.12; 73.72]	0.0%	2.5%
NCT00473889	1	124	0	124	3.02	[0.12; 74.96]	0.0%	2.5%
NCT00481078	4	63	0	32	4.92	[0.26; 94.19]	0.0%	2.9%
Krug et al. 2015	1	329	0	329	3.01	[0.12; 74.14]	0.0%	2.5%
Prebet et al. 2014	2	98	3	99	0.67	[0.11; 4.08]	15.2%	7.8%
Witta et al. 2012	3	65	0	63	7.11	[0.36; 140.54]	0.0%	2.9%
Ramalingam et al. 2010	10	62	2	32	2.88	[0.59; 14.05]	11.5%	10.2%
Jiang et al. 2019	15	244	0	121	16.41	[0.97; 276.64]	0.0%	3.2%
NCT00773747	20	315	11	320	1.90	[0.90; 4.04]	53.0%	45.0%
**Fixed effect model**		**2248**		**2058**	**3.12**	**[1.86; 5.23]**	**100.0%**	**--**
**Random effects model**					**2.32**	**[1.40; 3.84]**	**--**	**100.0%**

Heterogeneity: $I^2 = 0\%$, $\tau^2 = 0$, $p = 0.66$

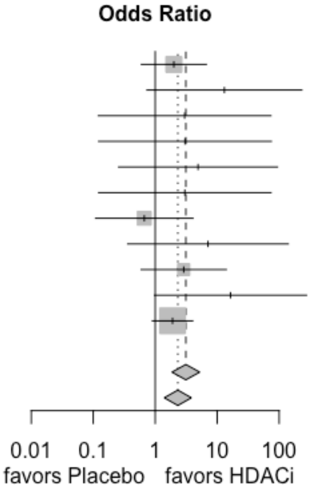

B. Change in Blood Pressure

Study	Experimental Events	Experimental Total	Control Events	Control Total	OR	95%-CI	Weight (fixed)	Weight (random)
San-Miguel et al. 2016	5	381	2	377	2.49	[0.48; 12.93]	15.7%	17.1%
NCT01802333	11	211	2	259	7.07	[1.55; 32.25]	13.5%	19.2%
NCT00473889	2	124	0	124	5.08	[0.24; 106.94]	0.0%	6.1%
NCT00481078	2	63	2	32	0.49	[0.07; 3.66]	20.3%	12.5%
NCT01933594	1	12	0	7	1.96	[0.07; 54.67]	0.0%	5.2%
Krug et al. 2015	3	329	0	329	7.06	[0.36; 137.30]	0.0%	6.4%
Prebet et al. 2014	3	98	4	99	0.75	[0.16; 3.44]	30.6%	19.1%
Ramalingam et al. 2010	3	62	2	32	0.76	[0.12; 4.81]	19.9%	14.4%
**Fixed effect model**		**1280**		**1259**	**2.28**	**[1.17; 4.44]**	**100.0%**	**--**
**Random effects model**					**1.84**	**[0.84; 4.04]**	**--**	**100.0%**

Heterogeneity: $I^2 = 19\%$, $\tau^2 = 0.2403$, $p = 0.28$

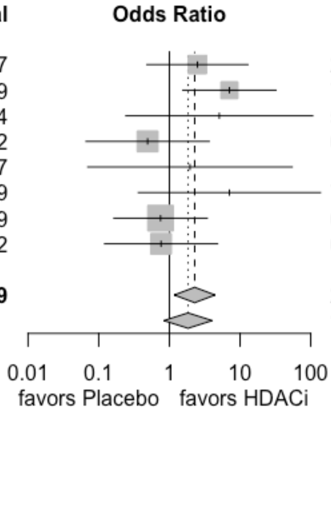

Fig S17: Forest plots of odds ratios (ORs) for datasets related to placebo/standard of care versus histone deacetylase inhibitor (HDACi). (**A**) hypokalemia, and (**B**) change in pressure (either hypo- or hyper-tension).
